# Genetic characterization of carrot root shape and size using genome-wide association analysis and genomic-estimated breeding values

**DOI:** 10.1007/s00122-021-03988-8

**Published:** 2021-11-15

**Authors:** Scott H. Brainard, Shelby L. Ellison, Philipp W. Simon, Julie C. Dawson, Irwin L. Goldman

**Affiliations:** 1 Department of Horticulture, University of Wisconsin-Madison, Madison, WI 53706 USA; 2 Vegetable Crops Research Unit, US Department of Agriculture–Agricultural Research Service, Madison, WI 53706 USA

## Abstract

**Key message:**

The principal phenotypic determinants of market class in carrot—the size and shape of the root—are under primarily additive, but also highly polygenic, genetic control.

**Abstract:**

The size and shape of carrot roots are the primary determinants not only of yield, but also market class. These quantitative phenotypes have historically been challenging to objectively evaluate, and thus subjective visual assessment of market class remains the primary method by which selection for these traits is performed. However, advancements in digital image analysis have recently made possible the high-throughput quantification of size and shape attributes. It is therefore now feasible to utilize modern methods of genetic analysis to investigate the genetic control of root morphology. To this end, this study utilized both genome wide association analysis (GWAS) and genomic-estimated breeding values (GEBVs) and demonstrated that the components of market class are highly polygenic traits, likely under the influence of many small effect QTL. Relatively large proportions of additive genetic variance for many of the component phenotypes support high predictive ability of GEBVs; average prediction ability across underlying market class traits was 0.67. GWAS identified multiple QTL for four of the phenotypes which compose market class: length, aspect ratio, maximum width, and root fill, a previously uncharacterized trait which represents the size-independent portion of carrot root shape. By combining digital image analysis with GWAS and GEBVs, this study represents a novel advance in our understanding of the genetic control of market class in carrot. The immediate practical utility and viability of genomic selection for carrot market class is also described, and concrete guidelines for the design of training populations are provided.

**Supplementary Information:**

The online version contains supplementary material available at 10.1007/s00122-021-03988-8.

## Introduction

Carrot (*Daucus carota* subsp. *sativus*) is a widely cultivated vegetable crop of both significant economic importance—globally, annual carrot production exceeds 40 million metric tons (FAO 2020)—and nutritional value, representing a significant source of pro-vitamin A in the human diet (Simon 2000). Carrot roots are sold into many different markets as a fresh product, a storage root, and a processing crop. In this regard, the size and shape of the edible, swollen taproot are key traits which not only influence yield, but are the principal determinants of market class in carrot (Banga 1957; Simon et al. 2008), affecting harvestability, post-harvest handling, and marketability. For example, processing industries (e.g., canning, freezing, dehydrating, or juicing) prefer to purchase cultivars that can produce a large, bulky root, which is typically grown as a long-season crop at relatively lower densities (500,000–1,000,000 plants per hectare). Fresh market uses, on the other hand, typically require longer, slimmer roots, which can therefore be grown at higher densities (1,500,000–3,000,000 plants per hectare).

While extensive diversity for root size and shape exists within cultivated carrot germplasm, these quantitative traits have historically been challenging to objectively evaluate. With the mechanization of carrot production, harvest, and post-harvest handling, these particular combinations of carrot root size and shape attributes have become increasingly important breeding targets. Nevertheless, distinguishing among market classes continues to be primarily performed based on a subjective visual assessment of the curvature of the carrot root shoulder and tip, as well as its length and width. No method for the quantification of standard size and shape categories is currently recognized. In this context, digital image analysis holds significant potential in not only automating phenotyping tasks, but enabling the precise measurement of the determinative components of market class.

Such an image analysis pipeline was recently developed specifically to provide a high-throughput method for accurately evaluating both size and shape parameters in a diverse collection of carrot germplasm (Brainard et al. 2021). This pipeline allows for the precise characterization of the morphological phenotypes which distinguish market classes from one another. In particular, principal components analysis (PCA)-based methods of quantifying shoulder and tip curvature, as well as size-independent variation in the full root contour, were shown to improve discrimination between market classes, relative to what is possible using only measurements of root length, width, and aspect ratio. The quantitative phenotypic data this platform provides, together with the recent construction of a high-quality, chromosome-scale reference genome for carrot (Iorizzo et al. 2016), allows researchers to now utilize both genome-wide association analysis (GWAS) and genomic-estimated breeding values (GEBVs) to analyze the genetic control of root shape in carrot. These methods are used widely in the study of plant genetics, due both to their ability to improve the efficiency of plant breeding, as well as provide a starting point for the molecular characterization of the genetic control of key agronomic traits.

This study utilized both of these methods, thus allowing for a methodological comparison of GWAS—which attempts to identify QTL through their non-random association with genetic markers—and GEBVs—which are based on an estimation of additive genotypic effects that does not rely on prior knowledge of QTL. Although GWAS has become a widely used tool in quantitative genetic analysis, even in cases where marker density is high and a heterogeneous diversity panel is utilized, small effect QTL often go undetected in the case of highly polygenic traits (Brachi et al. 2011). In contrast, GEBVs calculated using an infinitesimal model of gene action do not use any significance threshold for including markers in a predictive model. Since being initially developed in the context of animal breeding (Meuwissen et al. 2001), the development of efficient methods for calculating a marker-derived relationship matrix (VanRaden 2008) has led to the extensive use of GEBVs in agricultural breeding programs. Many of the factors that limit power in GWAS—such as population structure, population size, and trait heritability—can also limit the accuracy of such GEBVs. However, these methods have the potential to function in a complementary manner: while GEBVs have proven effective when performing selection even for highly polygenic traits, GWAS has the capacity to provide more specific information regarding the nature of the genetic control of traits, and the location of potential candidate genes.

To date, only two studies have examined the genetic control of carrot root size and shape (Turner et al. 2018; Macko-Podgórni et al. 2020), of which only one carried out GWAS analyses, and neither investigated the predictive ability of GEBVs for root shape traits. This paper therefore represents a novel advance in terms of our understanding of the genetic control of market class in carrot root, with implications both for further research and breeding for these traits. By combining quantitative measures of phenotypes extracted from digital images, with a diversity panel more than twice as large as that used in the only previously reported GWAS analysis, these results demonstrate the potential of association mapping in identifying QTL for root shape traits. In addition, these findings add support to the growing body of literature illustrating the utility of GEBVs for making selection on highly polygenic, quantitative traits, particularly in unstructured, outcrossing plant species.

## Materials and methods

### Plant materials

A total of 749 accessions (also referred to throughout this paper as “genotypes”) were utilized in this study, composed mainly of Plant Introductions in the USDA National Plant Germplasm System (USDA-NPGS) collection of *D. carota* germplasm held in Ames, IA—which includes open-pollinated varieties, inbred lines, and landraces—as well as breeding lines from both the University of Wisconsin and USDA-ARS carrot breeding programs in Madison, WI. This panel represented all cultivated carrots available in the USDA germplasm collection in 2016, and as such encompassed a wide range of root shapes, sampled from the majority of regions in which carrots are cultivated globally. A description of the geographic origin of each of the samples included in this analysis is included in Supp. File 1. Importantly, this panel excluded all 154 wild carrot samples utilized in the association analysis reported in Ellison et al. (2018), as these wild populations possess branched root systems, and thus are unsuitable for analyses focussed on market class traits.

In 2016 and 2018, the collection was grown at the Hancock Agricultural Research Station in Hancock, WI (44°08’N, 89°32'W); accessions were planted on May 16th and May 24th, and harvested on August 29th and 30th, respectively. In 2018–2019, the collection was grown at the University of California Desert Research and Extension Center in Holtville, CA (32°48’ N, 115°26’ W). In this environment, planting occurred on October 10th, and roots were harvested on February 25th. In both locations, accessions were grown in 1-m long rows; in Wisconsin, one replicate per genotype was planted, while in California, two replicates of all genotypes were planted in a randomized complete block design. One to fifteen roots were harvested per replicate. In Wisconsin, higher disease pressure led to fewer mature, undamaged roots being harvested per plot, on average. Following harvest, carrot tops were removed, and roots were stored at 4 °C until phenotyped.

### Phenotypic evaluation

Roots were digitally phenotyped as described by Brainard et al. (2021). In brief, after being cleaned, roots were QR coded and placed against either a white vinyl or black felt backdrop depending on root pigmentation. Images were acquired using a Nikon 5600 DSLR camera tethered to a computer running macOS 10.14. Python bindings for the OpenCV library were used to create binary masks of the roots by thresholding the hue-saturation-value color space. Custom MATLAB scripts were subsequently used to remove residual curvature in each root, and a random forest classifier was used to remove any unexpanded portion of the taproot. Python scripts for image acquisition and production of binary masks are available at: https://github.com/shbrainard/carrot-phenotyping; MATLAB scripts for straightening binary masks and performing PCA on contours or curvature values are available at: https://github.com/jbustamante35/carrotsweeper.

Following acquisition and pre-processing, phenotypes were extracted from the straightened, de-tipped binary masks. Root length was calculated as the distance from the center of the root crown to the root tip, following both straightening and elimination of the unexpanded, etiolated portion of the root. Maximum width was measured as the distance across the widest portion of the carrot, which is typically located just below the root crown. Total root size was defined as the 2D area of the entire binary mask. Aspect ratio was calculated as the ratio of length and maximum width. In addition, in order to quantify size-independent parameters of contour shape, PCA was performed on the root contour following a normalization procedure whereby each carrot was standardized to have a maximum width of 1, and a length of 1000. The scores along the first principal component quantify the degree of root fill, or how far down the length of the carrot the maximum width of the root is maintained; this trait accounts for over 80% of the variation in size-independent root shape. In addition, curvature values were computed at each point along the root contour in both the shoulder and tip regions as described by Driscoll et al. (2012). PCA of the first and last 50 elements of these curvature profiles was then performed; the first principal component of the former was used as a metric of shoulder broadness, while the first principal component of the latter was used as a measure of tip fill (a schematic workflow of this image acquisition pipeline is shown in Supp. Fig. 1). Together, this suite of root traits has been found to allow for accurate classification of roots, compared to a visual assignment of carrot market class (Brainard et al. 2021). In this study, two carrots were included in each raw image. With this workflow, the image acquisition phase required one minute per root, and one additional minute of computational time was required to perform pre-processing of the binary masks produced during acquisition, and phenotyping of these standardized images using a 3.3 GHz Intel Dual-Core i7 CPU and 16 GB of 2133 MHz LPDDR3 RAM.

Finally, prior to association analyses and construction of genomic prediction models, the diversity panel was restricted to those accessions that exhibited little to no branching of the taproot. Both the nature of the root-straightening algorithm—which depends upon identifying a carrot tip—and many of the phenotypes themselves (length, tip fill), implicitly require that the root be a single unbranched taproot. Small root hairs were removed through smoothing operations, and just as forked or split roots were discarded prior to image acquisition, accessions with highly branched fibrous root systems were also excluded on the basis of being inappropriate to an analysis of market class traits. Together with the failure of some roots to produce new leaf tissue following vernalization, this reduced the total size of the diversity panel used in subsequent analyses to 662 unique cultivated accessions.

### Estimation of genotype means

A “two stage” analysis was adopted in this study whereby prior to GWAS and genomic prediction, each genotype was first represented by a single phenotypic value, estimated using a mixed effects linear model. Genotype was modeled as a fixed effect, and each of the four unique combinations of location and replicate were combined into a single fixed “environment” effect with four levels. Because of unequal subsampling within environments, an additional random effect term was included to model genotype x environment interactions. The resulting model was equivalent to a RCBD model with subsampling:

$${Y}_{ijl}=\mu +{G}_{i}+{E}_{j}+{GE}_{ij}+{\varepsilon }_{ijl}$$

${G}_{i}$ represents the *i*th genotype effect, ${E}_{j}$ the *j*th environment effect, ${GE}_{ij}$ the *ij*th genotype x environment interaction (with ${GE}_{ij} \sim N(0, {\sigma }_{GE}^{2})$) and ${\varepsilon }_{ijl}$ the *ijl*th residual variation (i.e., variance among subsamples, with ${\varepsilon }_{ijl} \sim N(0, {\sigma }_{\varepsilon }^{2})$). Models were fit for each trait independently using the lme4 package in R v4.0.4 (R Core Team 2021), and genotype means were extracted using the package emmeans.

### DNA extraction, genotyping, marker development

Following six weeks of vernalization at 4 °C, one root per accession was transferred to a greenhouse environment and planted in conical tubes containing Pro-Mix High Porosity potting mix (Premier Tech, Quakertown, PA). Roots were maintained at 20 °C with a 16 h photoperiod. Following emergence of new leaf tissue, 1 cm^2^ leaf samples were obtained, and stored at − 80 °C until lyophilization. Freeze-dried tissue was then macerated, and genomic DNA was extracted using Macherey–Nagel NucleoSpin 96-well kits. DNA quantification (using Quantus PicoGreen dsDNA kits), library preparation, and sequencing was performed at the University of Wisconsin-Madison Biotechnology Center. In brief, restriction enzyme-digestion was performed with *ApeKI*, following which Illumina adapters and sample-specific barcodes were annealed. Samples were then multiplexed and sequenced on an Illumina NovaSeq 6000, generating on average 4 million, 150 bp paired-end reads per sample.

Raw, multiplexed.fastq files corresponding to forward and reverse reads of each lane were checked for quality, and demultiplexed using a custom a Java application (http://github.com/shbrainard/gbstools/). SNPs were then identified using the GBSv2 pipeline of TASSEL 5 (Bradbury et al. 2007), with version 3 of the *D. carota* genome (Iorizzo et al. 2016, 2020) used as a reference. Missing data was imputed with Beagle v5.1 (Browning et al. 2018), using default parameters, 20 iterations, and 300 phase states. Filtering, performed using bcftools v1.11, was used to remove markers with minor allele frequency less than 0.05, markers with 90th quantile depth less than 10 or greater than 500, and any non-biallelic markers. This filtering resulted in a total of 146,816 SNPs that were used as the basis for subsequent analyses. Genome-wide linkage disequilibrium (LD) was also calculated using bcftools, as the square of the sample Pearson correlation between marker genotypes (*r*^2^). Filtering on the basis of LD was performed using the prune plugin.

### Calculation of the realized-relationship matrix

For both GWAS and genomic prediction, SNPs were used to estimate a realized relationship matrix, ${\mathbf{A}}_{m}$, calculated using the imputed marker data:

$${\mathbf{A}}_{m}=\frac{\mathbf{Z}{\mathbf{Z}}^{\mathrm{T}}}{\sum_{k=1}^{m}2{p}_{k}{q}_{k}}$$

where $\mathbf{Z}$ represents a matrix of centered genotypes (662 accessions × 146,816 markers). The scaling factor insures diagonal elements are equal to $1+f$, where $f$ is equal to the intra-individual gametic correlation (Kang et al. 2008). ${p}_{k}$ and ${q}_{k}$ indicate the minor and major allele frequency for the ${k}^{th}$ marker. Shrinkage estimation was also applied in the case of estimating breeding values, using default settings of the A.mat function of rrBLUP v4.6.1, as described by Endelman & Jannink (2012).

### Linkage disequilibrium decay and population structure

LD was assessed in two ways. First, correlation coefficients between each SNP and its 100 nearest neighboring markers were calculated, and recorded along with the physical genetic distance between each pair. Distances were then binned, and LD regressed against genetic distance using the decay function $LD\left(x\right)\sim {y}_{f}+\left({y}_{0}-{y}_{f}\right){e}^{-{e}^{\left(log\alpha \right)x}}$, with initial estimates for ${y}_{f}$, ${y}_{0}$ and $log\alpha$ estimated in R using the self-starting regression function SSasymp. Second, genome-wide LD was visualized as a Manhattan plot by calculating the mean LD of each SNP with its 100 nearest neighbors, having first thinned the marker dataset to only 1 SNP per kilobase (kb), to avoid distortions due exclusively to uneven marker distribution across the genome.

Population structure was assessed by performing PCA on the centered marker matrix, and plotting the first two PCs against each other in a biplot. Scree plots of variance attributed to each component were also used to visually determine the number of PCs to include as fixed effects in the GWAS model.

### Genome-wide association analysis

GWAS was performed using the GWASpoly package (Rosyara et al. 2016), which implements the mixed model described by Yu et al. (2006). This tool utilizes the so-called *Q* + *K* method, whereby population structure and relatedness between individuals is controlled for using both fixed effects, as well as a random polygenic term calculated using all markers following the P3D method (Zhang et al. 2010). This resulted in the model:

$${\varvec{y}}=\mathbf{X}{\varvec{\beta}}+\mathbf{S}{\varvec{\tau}}+\mathbf{Z}{\varvec{u}}+{\varvec{\varepsilon}}$$

where ${\varvec{y}}$ is a vector of phenotypes, which in this study constituted the estimated genotype values from the linear mixed model described above. ${\varvec{\tau}}$ is a vector of SNP effects. ${\varvec{u}}$ is a vector of random polygenic effects, with a variance equal to ${\sigma }_{G}^{2}\mathbf{K}$, where ${\sigma }_{G}^{2}$ is the genetic variance, and **K** is proportional to the realized relationship matrix ${\mathbf{A}}_{m}$ defined above, but without scaling by $p$ and $q$. Because variance components were not re-estimated for each marker independently, this model is equivalent to that proposed by Kang et al. (2010). ${\varvec{\varepsilon}}$ is a vector of residual effects following a $N(0,{\mathbf{I}\sigma }_{\varepsilon }^{2})$ distribution, and ${\varvec{\beta}}$ is a vector of fixed population structure effects. $\mathbf{X}$, $\mathbf{S}$, and $\mathbf{Z}$ represent the respective incidence matrices. In this study, the first principal component of the marker matrix was used as a fixed effect, as proposed by Price et al. (2006) as an alternative to the groupings provided by clustering algorithms such as STRUCTURE (Pritchard et al. 2000).

Significance thresholds were determined on a trait-by-trait basis by performing 1000 simulated analyses using random permutations of each phenotype vector; this permutation testing was conducted using the computing resources and assistance of the UW-Madison Center for High Throughput Computing (CHTC) in the Department of Computer Sciences. Logarithm of the odds (LOD) thresholds were then calculated to control the family-wise error rate (FWER) at α = 0.05.

Partial *R*^2^ values and *p*-values for significant markers were calculated using backward elimination on the basis of a deviance parameter equal to the difference of likelihoods of the full model (with all significant markers included) and the reduced model (with the marker in question removed): $d=2({LL}_{f}-{LL}_{r})$, where ${LL}_{f}$ and ${LL}_{r}$ represented the log likelihoods of the full and reduced models, respectively﻿. For peaks in the Manhattan plots with multiple significant markers, single markers were identified by calculating, for each marker, *p*-values equal to the *d*th quantile of the cumulative distribution function of the χ^2^-distribution, with degrees of freedom equal to the number of SNPs in a given peak; peaks were then represented as the single SNP with a p-value < 0.05. Partial *R*^2^ values were then computed for these markers as: ${R}^{2}=1-{e}^{-\frac{d}{n}}$, where *n* represented the number of samples.

### Genomic-estimated breeding values

In addition to GWAS, marker data were used to calculate GEBVs, using best linear unbiased predictors (BLUPs) (Henderson 1963). First, the marker matrix used for estimation of kinship was thinned significantly: markers were thinned to a maximum density of one marker per 1 kb, with no missing data, resulting in 12,370 SNPs, with an average distance between SNPs of 36.55 kb; this marker density is sufficient to support maximal predictive ability, and thus was an appropriate base marker set for all subsequent analyses, as it allowed for each prediction accuracy-limiting variable to be evaluated individually. The ${\mathbf{A}}_{m}$ matrix was then calculated as above. BLUPs of the additive genotypic effects were then calculated using the rrBLUP package (Endelman 2011). The prediction error variance (PEV) of the BLUPs was calculated using the inverse of the coefficient matrix of the mixed model equations, scaled by the diagonal elements of the covariance structure defined by the realized relationship matrix (i.e., the variance of the given BLUP) (Henderson 1973); PEV is proportional to trait heritability, and thus provides a metric for judging the reliability of BLUPs without performing any form of cross-validation. Nevertheless, cross-validation of these predictions was also performed in order to assess predictive ability, by calculating the correlation of predicted values with an estimate of the true genotypic value. This was performed by first randomly masking 10% of the phenotypic data (the validation population; VP); correlation coefficients were then determined between the BLUPs of these genotypes calculated using the remaining 90% of the panel as a training population (TP), and their true phenotypic values. This was repeated 100 times, and average correlations were reported as predictive ability.

### Analysis of parameters affecting predictive ability

SNP density, the degree of relatedness between TP and VP, and TP size were all evaluated in terms of their effects on predictive ability using the cross-validation approach described above. In each case, the same self-starting regression function SSasymp used to model LD decay was fit to the resulting data. For any specific cross-validation analysis, all parameters not being varied were held constant at levels determined to not limit predictive ability.

For SNP density, VCF files were filtered according to a range of thinning parameters to generate variably dense marker sets. Markers were thinned to a maximum of one SNP every 0.1 kb (resulting in 18,093 SNPs, with an average distance between SNPs of 25.02 kb), 2 kb (resulting in 11,269 SNPs, with an average distance between SNPs of 40.04 kb), 5 kb (resulting in 6535 SNPs, with an average distance between SNPs of 47.11 kb), 10 kb (resulting in 4882 SNPs, with an average distance between SNPs of 56.68 kb), 100 kb (resulting in 2392 SNPs, with an average distance between SNPs of 181.17 kb), 250 kb (resulting in 1191 SNPs, with an average distance between SNPs of 361.97 kb), 500 kb (resulting in 660 SNPs, with an average distance between SNPs of 651.80 kb), 750 kb (resulting in 467 SNPs, with an average distance between SNPs of 926.63 kb), 1 megabase (Mb) (resulting in 362 SNPs, with an average distance between SNPs of 1.20 Mb), 2 Mb (resulting in 195 SNPs, with an average distance between SNPs of 2.26 Mb), 3 Mb (resulting in 136 SNPs, with an average distance between SNPs of 3.29 Mb), 4 Mb (resulting in 103 SNPs, with an average distance between SNPs of 4.35 Mb), and 5 Mb (resulting in 86 SNPs, with an average distance between SNPs of 5.27 Mb). A separate VCF file was also generated containing only markers on chromosome 3, thinned to a maximum of one SNP every 0.1 kb (resulting in 5621 SNPs, with an average distance between SNPs of 22.19 kb); this highly biased marker set provided an extreme case with which to evaluate the effects of distorting the genome-wide distribution of a relatively large number of markers.

To evaluate the effect of similarity between the TP and VP, a *k*-means clustering algorithm was applied to the principal components of the marker matrix containing 9535 SNPs. The most distant sub-grouping of 60 accessions was adopted as the most unrelated VP, and was then progressively diluted by replacing individuals in this sub-group with random draws from the larger population, to thereby simulate a gradient of relatedness between the TP and VP. Dilution amounts were set to 1, 3, 5, 7, 10, 13, 15, 17, 24, 27, 31, 35, 45, and 50 individuals, with 50 replications performed at each dilution level. “Distance” between TP and VP was then calculated on the basis of a similarity matrix, defined as the inverse of the distance matrix constructed from the first 100 PCs of the PCA of the marker matrix. Each individual in a given VP was then compared against the *n* most similar individuals in the TP, where* n* was allowed to vary between 1 and 660, depending on the distance metric being analyzed. These distances were then averaged across all individuals in the TP. The same 100-fold cross-validation approach as outlined above was repeated here for every TP/VP combination, and average predictive ability was then regressed onto the average similarity index.

To evaluate the effect of varying TP size, two distinct approaches were taken. First, absolute TP size was varied, while holding the relative size of the VP constant at 10%. This was performed by sampling subsets of sizes ranging from 10 to 660 from the full panel, repeating this sampling process 50 times at each population size, and performing the same 100-fold cross-validation approach as with previous analyses. Separately, relative TP size was also varied, by holding the absolute size of the VP constant at 60 individuals, and varying the total TP size from 75 to 660. As with the previous analysis, at each level of relative TP size, the sampling procedure was iterated 50 times, and for each iteration the 100-fold cross-validation approach was performed. Finally, relative VP size was varied, by holding the total population size constant at 662 individuals, and sampling VPs ranging from 10 to 650, and as above, iterating each VP size 50 times, and performing the 100-fold cross-validation with each iteration.

## Results

### Phenotypic variation in the diversity panel

**Fig. 1:**
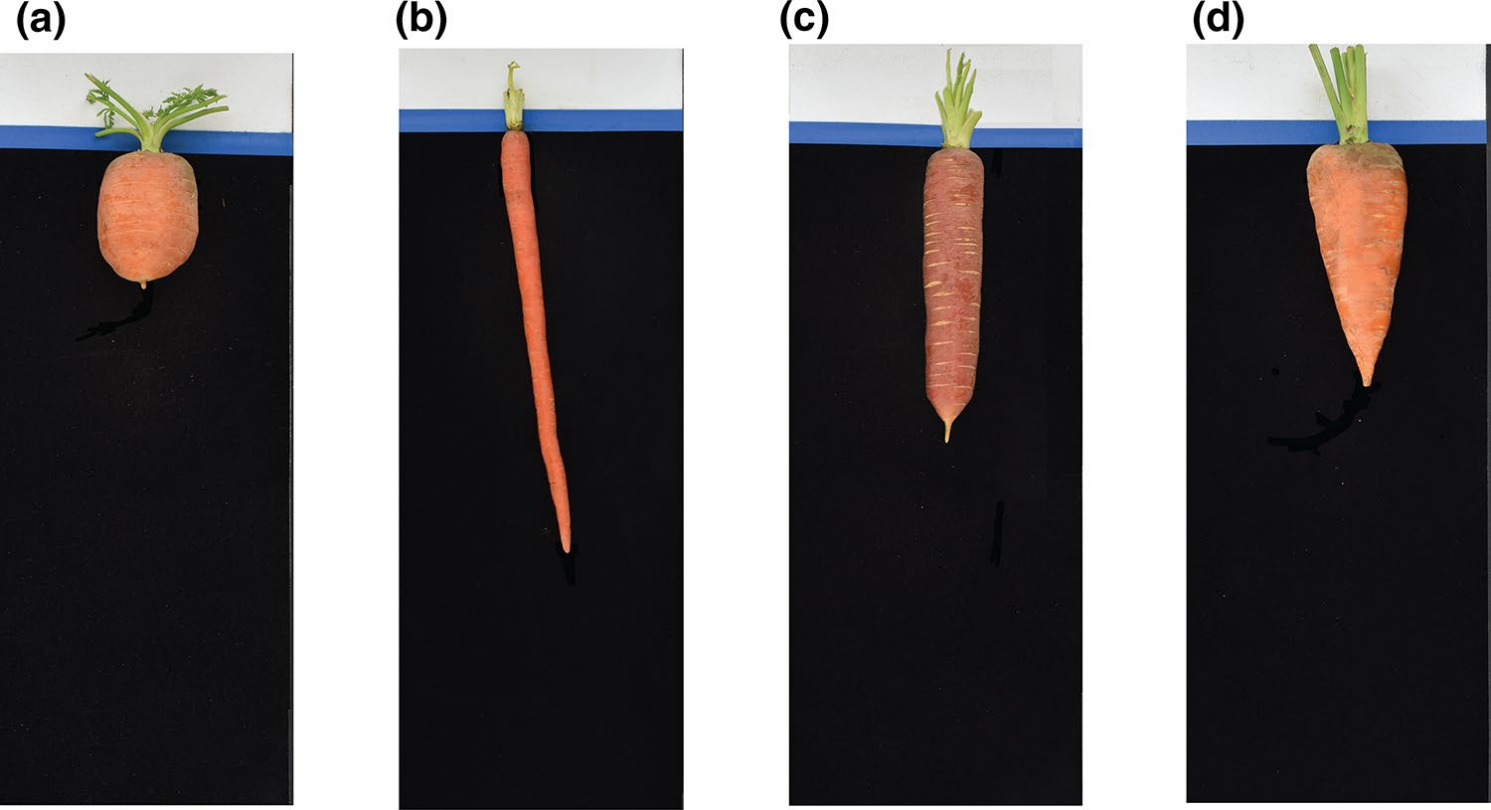
Representative roots from four distinct market classes, exemplifying variation in the four traits for which GWAS detected significant association with markers. **a** A Parisienne-type carrot and **b** an Imperator-type illustrate variation for length and maximum width. **c** A Nantes-type and **d** a Chantenay-type illustrate variation for aspect ratio and root fill

Representative roots drawn from four common market classes are shown in Fig. 1, illustrating the degree of phenotypic differentiation between classes, as well the particular combinations of the components of root size and shape which define specific classes. For instance, Imperator-type roots (Fig. 1b) combine a narrow maximum width with long root length, while the Chantenay-type (Fig. 1d) combines a large maximum width with low degrees of root fill; as described above, this latter trait reflects the first PC score following PCA on the size-normalized root contour. While all of the carrots shown in Fig. 1 clearly exhibit distinct aspect ratios, Fig. 1a-c all have high degrees of root fill, while only Fig. 1d exhibits a rapid tapering along its length. This highlights the particular value of image analysis procedures such as PCA, which allow for the de-coupling and independent analysis of size and shape parameters, and the extraction and quantification of high-dimensional phenotypes. Violin plots for each of these component market class traits, in each of the environments defined by a specific year and location combination, are shown in Supp. Fig. 2.

### Linkage disequilibrium and population structure

The extent and rate of decay of LD across the genome is an important determinant of the potential resolution of association analysis, and a decisive factor in determining the marker density necessary for performing GWAS (Otyama et al. 2019; Alqudah et al. 2020). A slow decrease in LD, as distance between pairs of markers increases, implies both that a relatively fewer number of markers is necessary to effectively capture the extent of historical recombination in the diversity panel, but also that large stretches of extended haplotype blocks will likely reduce the precision with which the size of a QTL can be estimated, due to long-range linkage between SNPs and causal loci (Myles et al. 2009).

**Fig. 2:**
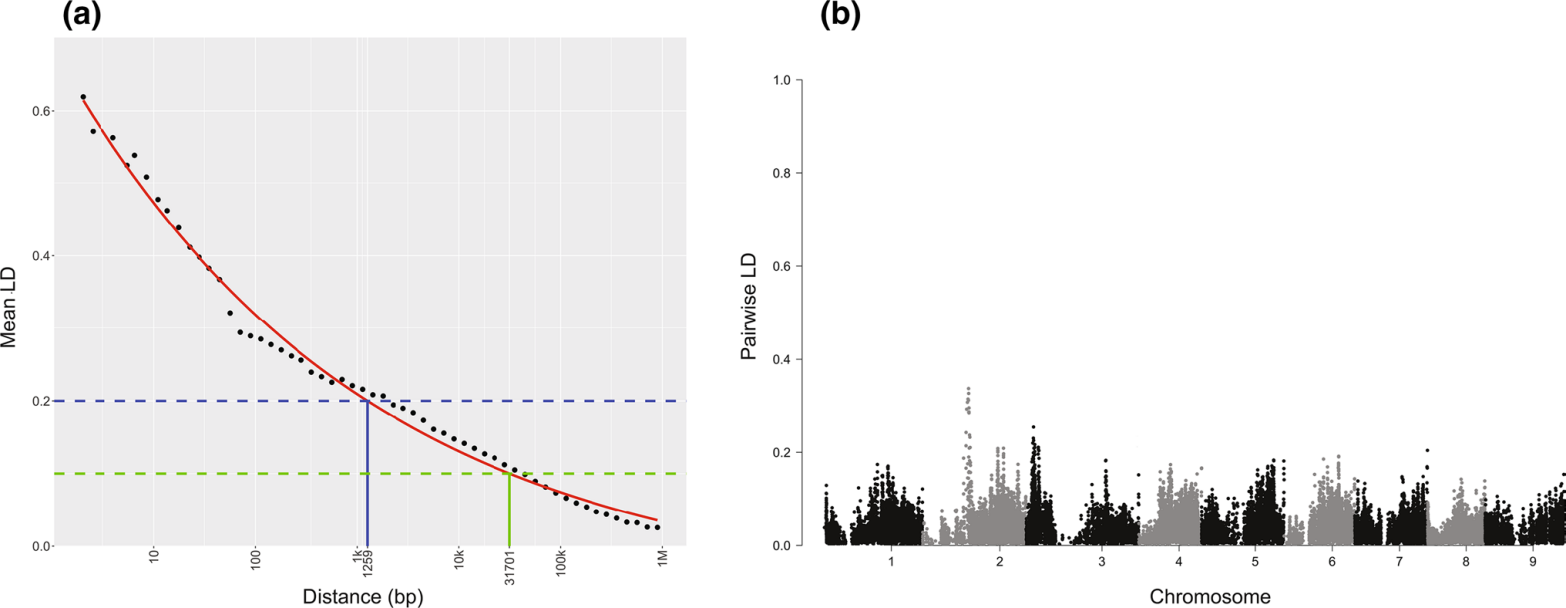
Genome-wide linkage disequilibrium in the diversity panel of 662 carrot accessions, using 29,456 SNPs represented in terms of a decay function (a), and Manhattan plot (b). **a** Average LD was plotted against the physical genetic distance between pairs of markers (black dots; log scale), and a self-starting asymptomatic decay function was fit to the data (red). Intercepts with *r*^2^ values of 0.2 (blue line) and 0.1 (green line) are indicated as 1259 and 31,701 bp, respectively. **b** LD calculated on a sliding-window basis (the mean of a given SNP and its 100 nearest neighbors) is represented as a Manhattan plot

Results of short-range LD decay are shown in Fig. 2a. Intersections with dashed lines representing *r*^2^ values of 0.2 (blue line) and 0.1 (green line) at 796 bp and 19.7 kb, respectively, illustrate that within only several kb, there is a rapid approach to linkage equilibrium across the panel. The curated marker set (with filtering parameters described above) contained SNPs separated by an average distance of 3711 bp; while this distance varied across the genome (Supp. Fig. 3), SNPs on average were therefore sufficiently close to provide adequate genome-wide coverage in terms of being in LD with putative QTL. Short-range genome-wide LD is visualized as a Manhattan plot in Fig. 2b. While a single peak in LD is observed on chromosome 2, average LD is relatively minimal, with a mean of only 0.038, and only 3.6% of all sliding windows exceeding the threshold of *r*^2^ > 0.1. This demonstrates both a consistent and relatively limited degree of LD across the genome. *r*^2^ values are accurate estimates of LD even using unphased genotypes (Rogers & Huff 2009), and thus the dense marker dataset utilized in this study appeared well-suited to association analysis.

**Fig. 3:**
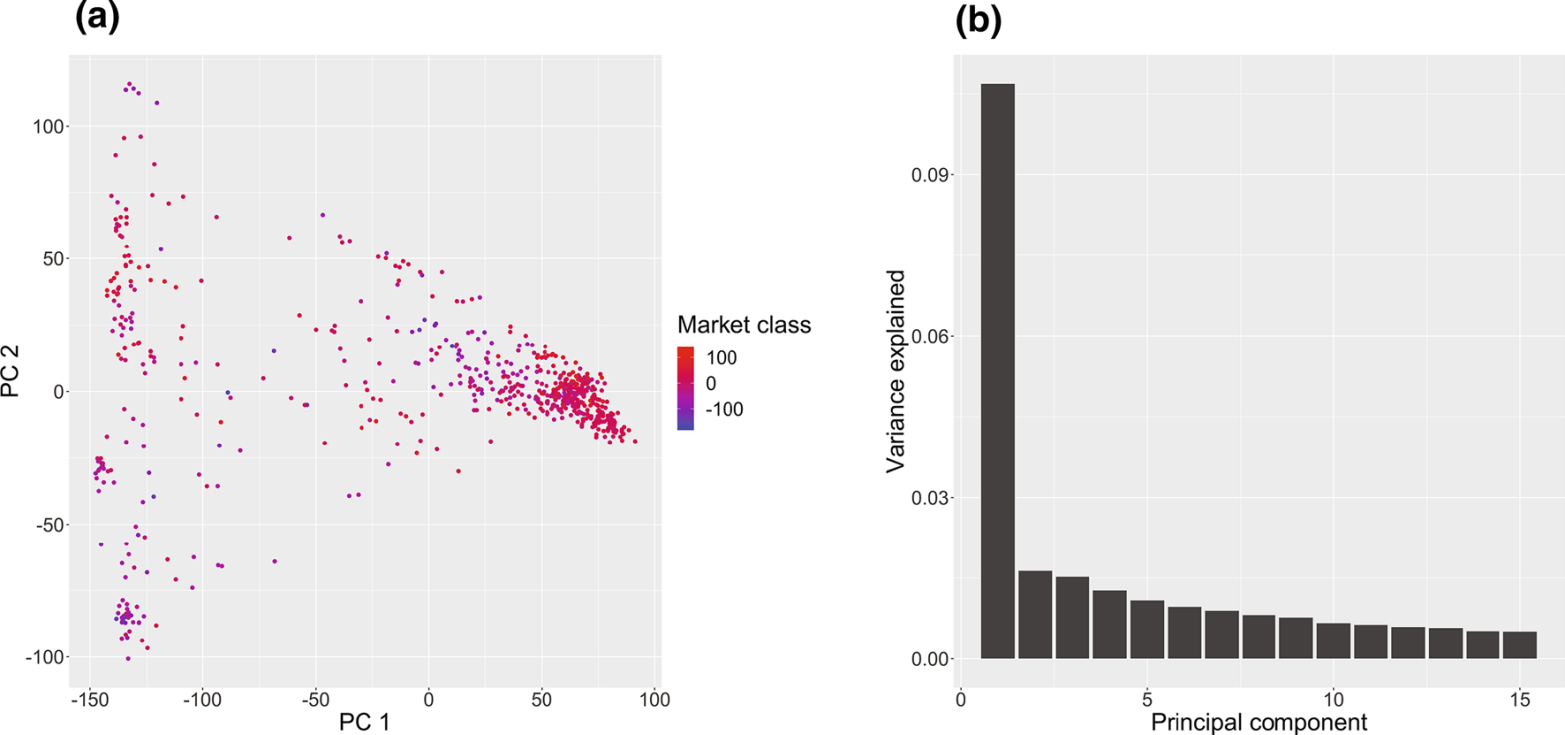
PCA-based visualization of population structure in the carrot diversity panel. **a** PCA bi-plot representing all accessions in the diversity panel according to their scores along the first (*x*-axis) and second (*y*-axis) principal component. Points are colored according to a quantitative measure of their market class following the methods of Brainard et al. (2021). This numeric score is itself calculated as the first principal component of a PCA on six market class traits—tip and shoulder curvature, root length, maximum width, aspect ratio, and root fill—and has previously been shown to effectively distinguish between the primary U.S. market classes. **b** Scree plot of the variance explained by the first 15 principal components of the PCA of the marker matrix

In addition to LD, which determines an upper bound on QTL resolution, and as such, informs appropriate marker density, population structure is another determinative characteristic of any association panel. The presence of uncontrolled population structure and admixture can lead to spurious inflation of *p*-values, even in the absence of severe linkage disequilibrium (Ewens & Spielman 1995; Pritchard & Rosenberg 1999). A PCA bi-plot was used to assess population structure, and the results mirror the minimal degree of structure observed by Ellison et al. (2018) (Fig. 3a). Aside from one primary cluster determined by scores along PC1 (which has been previously characterized as corresponding to the pool of Western domesticated carrot germplasm (Ellison et al. 2018)), little clustering was detected. And indeed, the variance captured by the first component was only ~ 10% of the total variance of the marker matrix, with each subsequent component explaining roughly 1% of total variance (Fig. 3b). Consequently, including one PC as a fixed effect in the association analysis was judged to be sufficient.

### Genome-wide association analysis

**Fig. 4:**
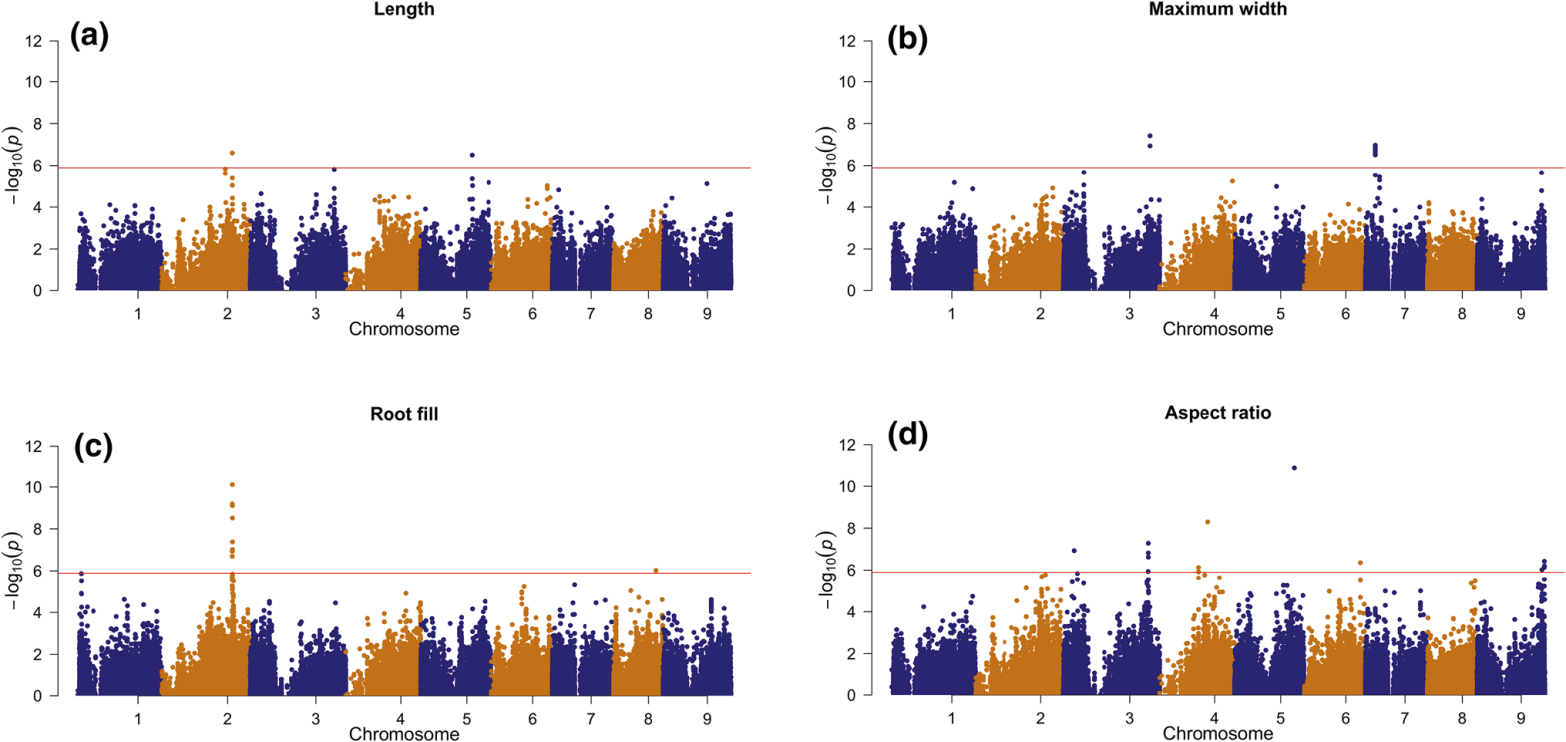
Manhattan plots of GWAS results using a diversity panel of 662 carrot accessions and 146,821 SNPs. Significant associations were found for 4 of the digitally-measured phenotypes quantified using the image analysis pipeline. **a** Root length; **b** Maximum width; **c** Root fill (the score along the first PC of the length- and width-normalized root contour; **d** Aspect ratio (length/maximum width)

Manhattan plots illustrating the results of GWAS for four root shape traits that are constitutive of market class are shown in Fig. 4. Three of these traits pertain specifically to the dimensions of the carrot root: length, maximum width (which occurs in the shoulder region of carrot roots), and their quotient, aspect ratio. These traits define the size of the root, and are in principle measurable by hand. In addition, significant associations were also found for root fill, which corresponds to the first PC score obtained by performing PCA on the length- and width-normalized root profile. This trait represents the most significant source of variation in the contour of the root—specifically, the extent to which a carrot maintains its maximum width down its length. Root fill is therefore explicitly a “shape trait”, insofar as it is a function of contours that have been standardized for their size, and as such, is not measurable by hand, though it reflects a key aspect of market class.

**Fig. 5:**
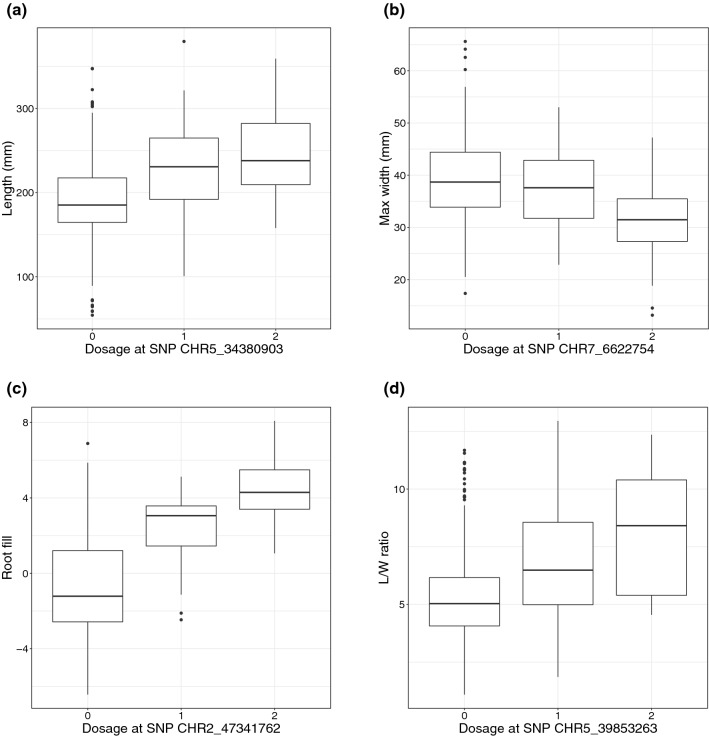
Box plots illustrating the effect of allelic substitution at individual significant SNPs for each of the traits shown in Fig. 4 (see Table 1 for LOD scores). **a** root length; **b** maximum width; **c** root fill; **d** L/W ratio. Integer values along the *x*-axis indicate the number of copies of the reference allele for the specified SNP

The most significant SNPs corresponding to each peak in a trait’s respective Manhattan plot are listed in Table 1, and box plots of the effect sizes of the individually significant SNP for each trait (represented as a function of allele dosage) are shown in Fig. 5. Annotated genes from version 3 of the carrot genome falling within a 40 kb window of the most significant SNP in each peak are listed in Supp. Table 1.

**Table 1:** Significant SNPs associated with root shape traits

Trait	Chromosome	Position (bp)	LOD score	Effect	*R*^2^
Root fill	2	47,341,762	10.11	− 0.91	0.06
Max width	3	58,042,921	7.42	− 1.78	0.04
	7	6,622,754	6.97	− 2.27	0.04
Length	2	42,684,849	6.60	− 15.28	0.03
	5	34,380,903	6.50	− 12.45	0.04
L/W ratio	3	7,295,883	7.06	0.64	0.01
	3	56,902,806	6.93	0.73	0.04
	4	26,702,749	6.12	0.65	0.04
	4	33,224,217	7.28	0.54	0.03
	5	39,853,263	10.90	1.13	0.07
	6	37,411,769	6.36	1.01	0.01
	9	45,393,500	6.42	0.80	0.02

Associations were found for four traits (root fill, maximum width, length, and aspect ratio) using a diversity panel of 662 carrot accessions genotyped for 146,816 SNPs, and phenotyped using the methods of Brainard et al. (2021). Chromosome, position, LOD score, and additive effect (relative to the reference allele) are listed for the most significant SNP in each peak exceeding the permutation test-derived ${-log}_{10}(p)$ threshold. *R*^2^ values are calculated on the basis of the difference in the log likelihood of the full and reduced models constructed using backward elimination of each of the significant markers for each trait

For three traits, relatively limited numbers of peaks were detected, with significant SNPs being located in a single peak for root fill, and only two peaks for length and maximum width, while for aspect ratio, seven peaks were detected. Effect sizes of these markers were relatively small, with partial *R*^2^ values ranging from 0.01 to 0.07 (Table 1, Fig. 5). Even in the case of aspect ratio, for which 7 peaks were detected, the *R*^2^ of the complete model was only 0.22. Two additional market class-related traits—shoulder and tip curvature—which reflect more subtle variation in the contour of these regions, were not found to be significantly associated with any SNPs (Supp. Fig. 4). Due to the relatively limited number of detected SNPs, and their small effect sizes, a number of additional GWAS models were tested to determine if the mixed linear model utilized here was overly conservative. However, the generalized linear model implemented in GAPIT (Lipka et al. 2012), known to generate fewer false negatives, led to substantial inflation (Supp. Fig. 5). The multi-locus mixed linear model implemented in GAPIT (Liu et al. 2016), led to results nearly identical to those shown in Fig. 4: all of the same peaks were detected using the MLMM, with equivalent LOD scores, although fewer significant markers were found within each peak (Supp. Fig. 6). The dominance model implemented in GWASpoly led to very similar results as well, although lower power was observed in several cases. Both of the peaks corresponding to QTL associated with root length were no longer detected, and three of the QTL associated with aspect ratio were also lost (Supp. Fig. 7).

### Genomic-estimated breeding values

PEV, average predictive ability, and minimum predictive ability are listed in Table 2 for all seven root traits which were digitally phenotyped in this study. While average predictive ability and PEV exhibited a wide range across these traits (0.25–0.86 and 0.35–0.92, respectively), it is clear that genomic predictions were able to provide reliable estimates of phenotypic performance for most of the components of market class. However, these average predictive abilities were calculated using 12,370 markers and the entire diversity panel, with 90% assigned to the TP. In applied contexts, where costs may limit both the size of training populations and marker datasets, it is of critical importance to understand how predictive ability changes as a function of these parameters.

**Table 2:** Prediction error variance (PEV), and predictive ability when the 100-fold cross validation was performed at random (Pred. ability—avg), and when relatedness between TP and VP was minimized (Pred. ability—min) for seven carrot root traits, phenotyped using roots grown across two locations and three growing seasons

Trait	PEV	Pred. ability—avg (sd)	Pred. ability—min
Total root size	0.71	0.67 (± 0.11)	0.29
Root fill	0.85	0.86 (± 0.03)	0.17
Max width	0.78	0.72 (± 0.06)	0.30
Length	0.80	0.77 (± 0.06)	0.29
L/W ratio	0.92	0.82 (± 0.05)	0.37
Tip fill	0.41	0.25 (± 0.04)	0.01
Shoulder curvature	0.35	0.63 (± 0.05)	0.10

#### i. Effects of marker density on predictive ability of GEBVs

**Fig. 6:**
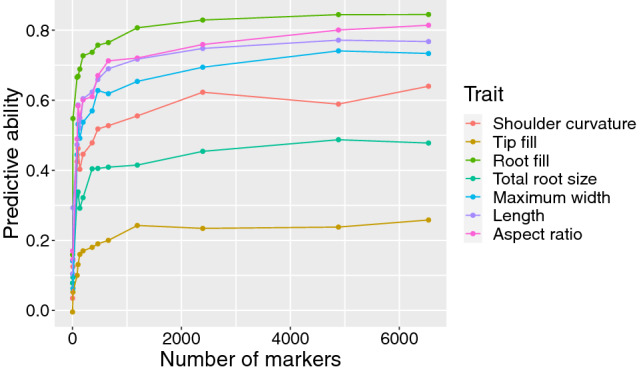
Effect of marker density on predictive ability for six root size and shape traits, as well as total root size, using the full carrot diversity panel. Curves follow an asymptotic exponential function, reaching their maximum at approximately 2500 markers

While the general result that increasing marker density increases predictive ability has been well-documented, the precise nature of the relationship will vary depending on the population and traits under consideration. Given that this diversity panel was genotyped at a high density for the purpose of GWAS, it was therefore feasible to generate marker sets generated through progressively more stringent filtering criteria, and thereby determine the effect of SNP density on predictive ability through cross-validation for each of the digitally phenotyped root traits evaluated in this study. The results of these analyses are shown in Fig. 6, and are well-described by an exponential function: at low marker densities, any increase in density is met with relatively rapid improvement in predictive ability. As marker density is increased, however, these improvements asymptomatically approach a maximum predictive ability, which in the case of these root traits is attained at roughly 2500 SNPs. This can be contrasted with GWAS, which, as described above, depends on a much denser array of markers across the genome in order to increase the likelihood that some subset of these will be in high LD with QTL. For example, when the marker dataset used in the GWAS analyses shown above was thinned to only 1 marker every 100 kb (i.e., 2392 markers), all significant associations between SNPs and QTL shown in the above Manhattan plots (Fig. 4) were no longer detected (Supp. Fig. 8).

Similar results were obtained by exclusively utilizing markers on a single chromosome, and comparing predictive ability against a random distribution of an equivalent number of SNPs. As shown in Supp. Table 2, utilizing exclusively markers on chromosome 3 only reduces predictive ability by an average of 12% across all traits. Some reduction in accuracy is to be expected, due to the relatively higher average degree of linkage between markers when all are located on a single chromosome. This finding is in line with those of Daetwyler et al. (2012), and highlights the fact that markers’ effect on predictive ability of GEBVs is primarily a function of their ability to accurately model covariance between individuals, and not their linkage with QTL.

#### ii. Effect of relatedness between the TP and VP on predictive ability

**Fig. 7:**
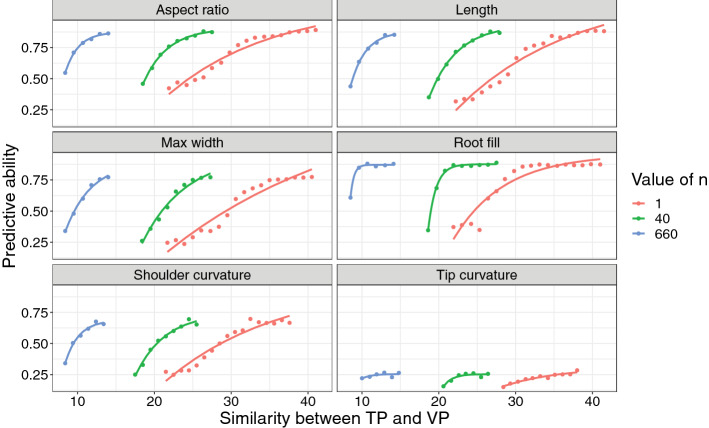
Effect of relatedness between TP and VP on prediction accuracy for the six key size and shape traits which constitute market class, using the full carrot diversity panel. Similarity is defined as the inverse of the Euclidean distance matrix, and *n* indicates the number of individuals in the TP against which each member of the VP is compared against

Increasing relatedness between the TP and VP has generally been found to be associated with improvements in predictive ability (Edwards et al. 2019; Olatoye et al. 2020). This was corroborated here by regressing predictive ability onto three different measures of relatedness (i.e., three different values of *n*, as described above), as shown in Fig. 7. For all traits, an exponential regression similar to that utilized in the case of assessing SNP density was performed. Prediction accuracies were substantially reduced from their maximum when similarity between the TP and VP was minimized, and as similarity increased, predictive ability increased exponentially, approaching a maximum that itself was the average predictive ability reported above. This convergence to the mean can be understood as a consequence of the fact that this random sampling procedure will on average select a VP that is extremely similar to the TP for this diversity panel, due to the minimal degree of population structure.

In addition to this general trend, it is also clear that across all traits, both extremely high and extremely low values of* n*—i.e., the number of individuals in the TP that each member of the VP was compared against—are more poorly modeled using the exponential regression although for distinct reasons. In the case of the former, due to oversampling, the possible range of similarities between the TP and VP is significantly compressed toward low values. Because average prediction accuracies are high, this has the additional result of inflating prediction accuracies at all levels of similarity. At the other extreme, when *n* = 1, there is clearly substantial noise around the exponential regression, which is a consequence of this metric of similarity providing an inaccurate representation of the overall similarity between the TP and VP due to under sampling; that is, more than one individual must be similar to each individual in the VP in order to make accurate predictions. While these results do not provide a basis for determining a specific value of* n* that should be used for any arbitrary TP/VP combination, they do justify intermediate values of* n* (e.g., in this study, 40) that are both highly precise in that they fit the exponential regression function extremely well, while also being highly informative, in that they facilitate discriminating between degrees of relatedness for a wide range of TP/VP combinations.

#### iii. Effect of population size on predictive ability

**Fig. 8:**
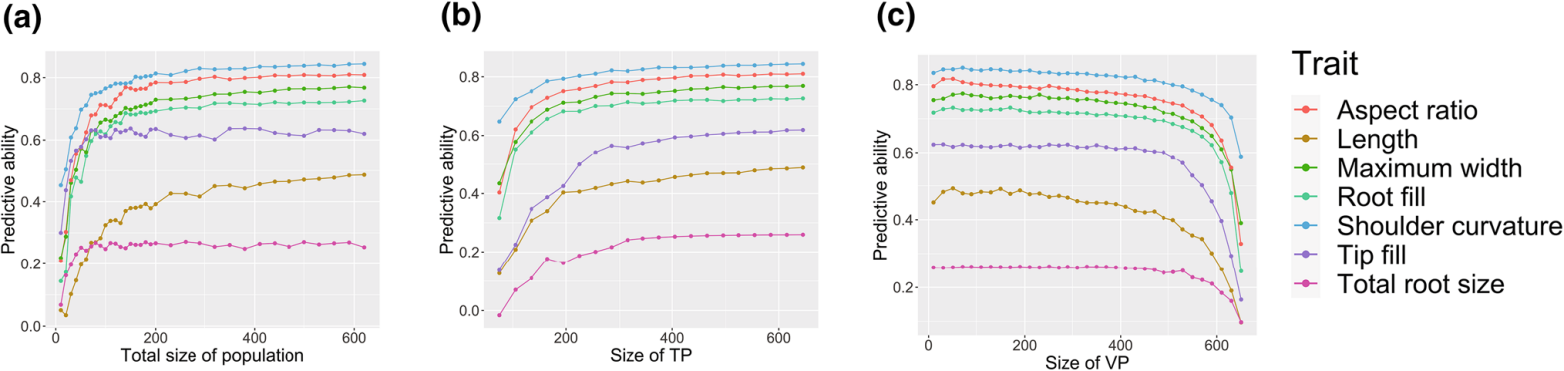
Effect of population size on prediction accuracy for the six traits underlying carrot market class, as well as total root size. **a** Absolute population size is varied, holding the VP at 10% of the total population; **b** The relative size of the TP is varied by holding the VP constant at 60 individuals; **c** Relative VP size is varied by keeping total population size constant at 662 individuals

The third parameter evaluated in terms of its effect on predictive ability was the size of the TP. There are three distinct ways in which the effect of TP size can be modified, as described above: absolute population size can be varied, relative TP size can be varied, or relative VP size can be varied. First, the effect of varying absolute population size, with VP size held constant at 10%, was evaluated. For all traits, predictive ability reached its asymptote at roughly 330 genotypes (Fig. 8a). Similarly, when the VP size was held constant at 60 individuals and relative TP size is varied, all traits appeared to follow a similar dynamic, with predictive ability attaining its maximum again at roughly 330 genotypes (Fig. 8b). Finally, the effect of varying relative VP size, with the TP held constant at the total population size of 662 genotypes (less the size of the VP) was analyzed. Again, all traits followed the relationship seen in the previous two cross-validations: across all traits, predictive ability reached its asymptote when the TP was less than roughly 50% of the total population, or 330 genotypes (Fig. 8c).

## Discussion

This study identified a novel set of QTL for four of the most relevant morphological components of root market class in carrot. This included a highly significant SNP on chromosome 2 associated with root fill; as such, this represents the first genetic characterization of the control of the vast majority of the variance in root shape. For three of these four traits, only two QTL per trait were identified, and their effect sizes were relatively small, ranging from 0.01 (for the SNP on chromosome 6 associated with aspect ratio) to 0.06 (for the SNP on chromosome 2 associated with root fill), with total phenotypic variance explained ranging from 0.06 for root fill to 0.22 for aspect ratio. This is surprising, given the relatively high heritabilities observed for these phenotypes, both as estimated here using genomic data, and as previously reported using a diallel mating design (Turner et al. 2018; Brainard et al. 2021). This would suggest that the effect sizes of the identified molecular markers is being underestimated, or that there are additional unidentified QTL, or both.

There are multiple possible explanations for these possibilities. First, as described above, carrot is a highly heterozygous outcrossing species, and indeed, most of the accessions included in this diversity panel are not inbred lines, but are instead landraces, open-pollinated varieties, and populations. One consequence of this was the strikingly rapid decay in linkage disequilibrium within this diversity panel, with an average *r*^2^ of 0.2 between pairs of markers obtained within a distance of only 796 bp. Despite the dense marker distribution used in this study, therefore, it is likely that this rapid decay of LD led to an underestimation of the effect size of the molecular markers that were identified in this study, as well as the number of QTL themselves. Further complicating this analysis is the fact that selection for root shape morphology has likely occurred in numerous genetic backgrounds, with multi-allelic combinations producing similar root shapes within, e.g., Western European, Eastern European and North American accessions, and the USDA-NPGS collection used in this study included accessions from all of these geographic regions. In this regard, linkage mapping could provide a fruitful subsequent line of analysis, by addressing the under-estimation of effect sizes due to differences in frequencies between marker alleles and QTL alleles.

Despite these limitations, a number of candidate genes were identified on the basis of these analyses, and are listed in Supp. Table 1. While these gene models are the product of in silico-based annotation, most have predicted functions based on homology to the peptide sequences of known gene families. In particular, for three of the four market class traits for which QTL were identified, candidate genes were identified which have been previously described to play a role in root development processes. For length, the gene DCv3_Chr5.21023, located in the peak on chromosome 5, is a predicted piezo-type mechanosensitive ion channel, which has been implicated in the capacity of roots to grow vertically through the soil profile (Mousavi et al. 2021). For aspect ratio, a gene in the peak on chromosome 9, DCv3_Chr9.36166, is a homolog of protein terminal *ear1*, which has been associated with abscisic acid-mediated root growth (Wang et al. 2018). And for root fill, three genes were identified in the highly significant peak on chromosome 2: DCv3_Chr2.08059, which encodes a homodomain-leucine zipper (HD-Zip) protein that has been linked to root development (Elhiti & Stasolla 2009); DCv3_Chr2.08061, which is a homolog of non-DNA-binding bHLH transcription factors that are involved in lateral root formation (Castelain et al. 2012); and DCv3_Chr2.08063, which is homologous to a AAA-ATPase protein found in *Arabidopsis thaliana* that has been found to drive adventitious root formation (Xu et al. 2018a, b). Because of the highly crop-specific nature of the market class phenotypes evaluated in this study, it is unsurprising that the putative functions of these genes do not overlap precisely with the corresponding carrot traits studied here. Nevertheless, these genes all represent viable candidates for further investigation to elucidate a precise mechanistic model of the molecular pathways underlying root size and shape variation in carrot.

Tip fill (e.g., the blunt-tipped Nantes-type in Fig. 1c vs. the pointed Chantenay-type in Fig. 1d) and shoulder broadness (e.g., the highly curved Parisienne-type in Fig. 1a vs. the straight-shouldered Imperator-type in Fig. 1b) represent more subtle aspects of root shape variation, since they are restricted to specific regions of the root contour. Though evidently important in distinguishing between market classes, no SNPs were found to be significantly associated with phenotypic variation for these two traits. In addition, for the components of market class for which QTL were identified, the significant SNPs explained a small percentage of the total variation for these traits. This is not an uncommon result when the traits under consideration are quantitative and highly polygenic, as would appear to be the case here. Indeed, it is consistent with the only other published report of a GWAS that included carrot root traits (Macko-Podgórni et al. 2020), which reported QTL on chromosome 1 that accounted for roughly 10% of the phenotypic variation in maximum width. The panel utilized by Macko-Podgórni et al. (2020) differed significantly from the material used in this study, representing 103 accessions from the Warwick Crop Centre in Wellesbourne, UK. It is therefore not surprising that the QTL identified in that genetic background was not detected in our analysis, but nevertheless, the low effect size is consistent with that reported here. Similarly, a previous study that attempted to use linkage mapping to detect QTL for carrot root traits detected no QTL for width or aspect ratio, and three QTL associated with length which each explained less than 10% of the phenotypic variation (Turner et al. 2018). These results are not directly comparable with those presented here, since this population was an F_2_ family descended from a cross between only two accessions, and thus represented a more limited range of genotypic and phenotypic diversity. Nevertheless, the low effect sizes of the QTL are consistent with this study's findings.

The predictive ability of GEBVs was evaluated using the same diversity panel and marker set used in the GWAS analysis, so as to allow for an accurate assessment of their potential complementarity. Interestingly, for all traits except for total root size, average predictive ability was quite high; this was true even of shoulder broadness and tip fill—traits for which no QTL were identified via GWAS. This is not entirely surprising, due to the fact that GWAS relies on detecting significant associations between markers and QTL, while GEBVs are simply the additive genotypic effects predicted using markers as the basis of a covariance matrix for modeling relatedness. In order to assess how robust these predictive abilities would be given various marker densities, population sizes, and degrees of relationship between training and validation sets, cross-validation analyses were performed.

Regarding marker density, the asymptote of the exponential relationship between SNP density and predictive ability was attained a relatively low number of markers. In the case of GWAS, utilization of a large diversity panel which contains a high amount of historical recombination necessitates the use of a dense array of molecular markers across the genome, in order to maximize LD between markers and QTL. Compared to the 146,816 markers used in the GWAS analysis, however, maximum predictive ability of GEBVs was attained with only several thousand markers. This is consistent with the ranges presented in previous studies (Erbe et al. 2013; Wang et al. 2017; Wu et al. 2016; Zhang et al. 2015), and highlights the different role that markers play in an analysis based around testing significant associations (particularly in a species with rapid LD decay) versus estimating genomic relatedness.

These results demonstrate the manner in which, from a practical perspective, the genotyping costs associated with implementing genomic selection are at least in principle less than those associated with GWAS, though in practice this would depend on a high-quality genotyping platform that generated only thousands, instead of hundreds of thousands of markers. In addition, it is important to note that loci which were called as heterozygous in the marker dataset used here are segregating in the corresponding accession, and utilizing the genotype of a single root will necessarily mask this intra-accession variation. Techniques such as PoolSeq, which utilize bulked DNA from multiple individuals for GBS sequencing, have been found to be potentially useful in such situations (Anand et al. 2016; Bélanger et al. 2016). By sequencing at a high depth, it is possible to utilize continuous measures of allele frequency in the linear mixed model used to test for associations between SNPs and a given phenotype (instead of the categorical allele dosages used here).

With respect to population structure, because predictions are based on a covariance matrix relating phenotyped individuals to non-phenotyped individuals, higher degrees of relatedness between the TP and VP typically lead to higher predictive ability. However, this phenomenon has historically been investigated qualitatively, by either comparing prediction accuracies across populations with known degrees of variable relatedness (such as full- vs. half-sib families), or between population groupings defined through clustering algorithms such as STRUCTURE (Riedelsheimer et al. 2013; Sverrisdóttir et al. 2018; Lozada et al. 2019). While it would be theoretically feasible to apply a clustering algorithm to the panel presented in this study, this approach would be of limited utility given this panel's limited degree of population structure. Because this population is not composed of well-defined, discrete subpopulations, more relevant is the relationship between a quantitative measure of the degree of relatedness between the TP and VP, and predictive ability.

Furthermore, in cases where relatedness has been measured between the TP and VP quantitatively, the appropriate metric has been assumed to be a comparison of the means of the two groups—i.e., the measure obtained by setting the value of *n* to the size of the TP, and thus comparing each individual in the VP against each individual in the TP (e.g., as in Berro et al. (2019)). The cross-validations performed in this study therefore represent an advance in terms of the precision with which conclusions regarding the effect of relatedness on predictive ability can be made. Not only was increasing similarity between the TP and VP associated with improvements in predictive ability in this study, this relationship was well-described for most traits by the same exponential function utilized in the case of marker density. In addition, intermediate values of* n* were found to give the most precise and informative measure of similarity in terms of defining this exponential relationship. Finally, regarding conclusions one can draw about this particular diversity panel, it is clear that one of the factors contributing to high prediction accuracies on average is that the mean level of relatedness between a randomly selected TP and VP is extremely high. Measured with* n* = 40, average similarity was 23.1, with a standard deviation of 0.56, which is already in the range of relatedness that defines the asymptotic portion of the exponential function, and thus it is reasonable to assume that these GEBVs would be robustly accurate to any arbitrary construction of TP and VP, given a breeding population similar in structure to the diversity panel analyzed here. In this regard it is important to emphasize that while this diversity panel is relatively unstructured, and contains a large amount of genetic variation, breeding populations often exhibit high levels of overall relatedness among individuals, clear structure due to defined pedigrees, and significantly less phenotypic diversity than a global germplasm collection. The results described here, therefore, will not be necessarily transferable to every specific breeding context.

It is interesting to note that not all traits exhibited an exponential relationship between predictive ability and relatedness equally well. Certain traits, such as root fill and aspect ratio, clearly followed this exponential relationship (Fig. 7, Supp. Fig. 9). Others, such as shoulder curvature, tip fill, and maximum width displayed a more linear relationship. This variation in the fit of the exponential regression is orrelated with the maximum predictive ability attained for each of the traits, and therefore would suggest that the asymptotic portion of the relationship is only evident when high predictive ability is attainable, given a wide range of simulated similarity levels.

Finally, the effects of TP size were considered in this study by explicitly considering two distinct cases. First, the consequence of varying absolute population size was evaluated. Second, the effect of changing the relative size of either the TP or the VP was analyzed. These effects typically are confounded with each other in studies that have examined how to optimize the size of TPs: Xu et al. (2018a, b) considered only the effect of varying the absolute size of the TP, by maintaining the VP at 20% of the total population size; Tayeh et al. (2015) considered only the effect of varying the relative size of the TP, by holding the absolute VP size constant, and varying the size of the overall population; Zhang et al. (2017) varied both the absolute size of the TP, and the relative size of the VP at each of these levels, but averaged across all of the relative size variations, reporting only the effect of changes in absolute TP size.

In the cross-validations reported here, in all three cases, an exponential relationship was found between predictive ability and either the total size of the population, or the relative size of the VP or TP. While it is unsurprising that increasing the total number of individuals in the panel would increase predictive ability, it is interesting to note that in the case of varying relative TP size, (either by increasing the size of the TP while holding the VP constant, or conversely by decreasing the size of the VP while holding the TP constant), the key determinant was primarily the number of individuals in the TP, scaled by the number of individuals for which one is attempting to predict performance. It is also interesting to note that the point at which the asymptotic maximum predictive ability is attained—roughly 50% of the total diversity panel—is consistent with reports of the minimum TP size needed to attain maximum predictive ability in squash (Hernandez et al. 2020), and carrot (Corak et al. 2019; Corak 2021).

While the results of these cross-validations are similar—i.e., increasing the number of individuals in the TP leads to an increase in predictive ability—they clearly differ in their precise interpretation, and most importantly, answer very different practical questions from a resource allocation perspective. For instance, if genotyping costs are most limiting, it may be more critical to know the minimum total population size needed to obtain the asymptotic maximum, or a minimum desired, predictive ability for a given trait; this would correspond to analyses which vary absolute population size. This can be contrasted with a scenario in which phenotyping costs are most restrictive for a specific trait, and it is therefore more relevant to consider the minimum useful TP size, since the aim would be to predict the performance of a maximum number of non-phenotyped individuals; this in turn would correspond to analyses which vary relative TP size. Finally, if the total pool of germplasm available is predefined, as in the case of a genetic resources collection, it may be most important to consider the minimum proportion to phenotype without sacrificing predictive ability; this, logically, corresponds to scenarios which vary the relative size of the VP.

It is also relevant to note the general pattern observed for the cross-validations of the predictive ability of GEBVs reported here: i.e., the exponential relationship between predictive ability and the variable under consideration. Importantly, asymptotic maximum predictive ability was reached at low values of SNP density, population relatedness and population size, relative to the total marker density, level of population structure, and diversity panel size in the carrot collection presented in this study. From a practical perspective, it therefore appears very tenable to attain non-limiting levels of nearly all the determinative factors influencing predictive ability.

### Practical comparisons between GWAS and GEBVs

While GWAS offers a preliminary method to gene discovery, and the development of marker-assisted selection breeding strategies, genomic selection offers the potential for the more immediate use of marker information by predicting additive genotypic effects based on relatedness (Minamikawa et al. 2018; Srivastava et al. 2020; Tsai et al. 2020). In addition, however, GWAS is poorly suited to the detection of numerous minor effect QTL that underlie quantitative traits (Robinson et al. 2014; Caballero et al. 2015). Even when QTL for highly polygenic traits have been previously detected through interval mapping approaches, marker-assisted selection based on multiple linear regression using QTL-linked markers has been observed to have lower prediction accuracy than genome-wide prediction models (Hadasch et al. 2016). In this context, a clear conclusion from the results presented here is that the traits which underlie market class in carrot are certainly highly polygenic: the robust, high predictive abilities described above are an undeniable function of the large additive genetic components controlling these root phenotypes, while the limited number of small-effect QTL detected via GWAS reflect the numerous small effect loci which therefore underlie this additive variance.

Finally, it is relevant to note the practical implications for the mode of genomic selection that would be enabled on the basis of the predictions made in this study. As indicated in the name “genomic-estimated breeding values”, what has been estimated in this study are explicitly the additive components of genotypic value, and as such, the portion of a given individual accession’s value that is transmissible to the next generation. The immediate practical utility of such predictions most likely falls within population improvement efforts. In particular, the most frequent use of diversity panels such as the one utilized in this study is the identification of novel traits that currently are not present in elite germplasm. Through the introgression of such traits into breeding lines, market class attributes would likely be impacted; the GEBVs reported here could therefore significantly accelerate the pace at which a particular desired market class is recovered, following such wide crosses. Despite their promise, the actual gains from selection one can expect to attain will vary from trait to trait. While some phenotypes presented here, such as root fill, length, and maximum width, can be predicted with notably high accuracy, others, such as total root size and tip fill, are markedly more difficult to predict. This is unsurprising, since root size is clearly a composite trait, much like yield. Variations in more subtle shape traits, e.g., tip fill, will for their part, likely always be subject to greater environmental variation, and thus be challenging to select for, particularly when genotypes are harvested following a fixed number of days following planting. Nevertheless, despite the lower prediction accuracies for these traits, GEBVs still offer a method for utilizing genomic-scale data to aide in improving the efficiency of selection. Given the high-throughput nature of the phenotyping platform which could be used to collect data on a training population, and the relatively limited amount of sequencing required to calculate GEBVs, this study provides compelling evidence supporting the inclusion of genomic selection in breeding programs for carrot market class. We hope the extensive cross-validation analyses presented here are able to provide concrete direction for research groups attempting to implement genomic selection protocols within their own breeding programs.

## Supplementary Information

Below is the link to the electronic supplementary material.

Supplementary file1 (DOCX 26 kb)

Supplementary file2 (DOCX 19 kb)

**Supplementary Fig. 1** Image acquisition workflow (adapted from Brainard et al. 2021). **A** Each black-bordered box within the overall image is first identified; **B** QR codes in the upper portion of each sub-box were scanned; **C** Carrot pixels were differentiated from background pixels to generate binary mask images; **D** Binary masks were saved to disk according to a file structure and naming scheme based on the information encoded within each QR code. **E** The midline of the carrot root was estimated by tracing a path from the carrot tip to the center of the shoulder, following the maximum of the smoothed Euclidean distance transform; **F**dth measurements were made by sampling the binary mask normal to vectors tangent to points along the midline; **G-H**A random forest classifier was used to detect the point at which to “de-tip” any residual, unexpanded portion of the tap root

**Supplementary Fig. 2** Histograms, represented as violin plots for each of the three carrot root production years, for the 6 component market class traits

**Supplementary Fig. 3** Distribution of the SNP marker dataset used in GWAS analyses. Genome-wide coverage is shown for each of the 9 carrot chromosomes

**Supplementary Fig. 4** Manhattan plots for GWAS results for tip fill and shoulder curvature, using a diversity panel of 662 carrot accessions and 146,821 SNPs

**Supplementary Fig. 5** QQ-plots of *p*-values generated by the generalized linear GWAS model implemented in GAPIT, using the same diversity panel of 662 carrot accessions and 146,821 SNPs as used in the mixed linear models

**Supplementary Fig. 6** Manhattan plots generated using the multi-locus mixed linear model (MLMM) GWAS model implemented in GAPIT, using the same diversity panel of 662 carrot accessions and 146,821 SNPs utilized in the other GWAS analyses

**Supplementary Fig. 7** Manhattan plots generated using the dominance GWAS model implemented in GWASpoly, using the same diversity panel of 662 carrot accessions and 146,821 SNPs utilized in the other GWAS analyses

**Supplementary Fig. 8** Manhattan plots of GWAS results using a diversity panel of 662 carrot accessions and only those SNPs located on chromosome 3. No significant associations were found for four root traits for which associations were previously detected (**a** root length **b** maximum width; **c** root fill and **d** aspect ratio)

**Supplementary Fig. 9** Prediction accuracy versus population structure at a variety of values of *n*, illustrating the variable goodness of fit of the asymptotic exponential function for 6 traits: **a** total root size; **b** length; **c** root fill; **d**aspect ratio; **e** maximum width; **f** tip curvature. Phenotypic data from the entire diversity panel of 662 carrot accessions was used in all analyses

Supplementary file12 (CSV 38 kb)

## Data Availability

SNP markers, as well as binary masks of the images used for phenotyping are available via the Harvard Dataverse: https://dataverse.harvard.edu/dataverse/usda-npgs-carrot-collection.

## Code availability

Links to the source code for the digital image-based phenotyping platform are provided in the “Methods” section.

## References

[CR1] Alqudah AM, Sallam A, Stephen Baenziger P, Börner A (2020). GWAS: Fast-forwarding gene identification and characterization in temperate Cereals: lessons from Barley: a review. J Adv Res.

[CR2] Anand S, Mangano E, Barizzone N (2016). Next generation sequencing of pooled samples: guideline for variants’ filtering. Sci Rep.

[CR3] Banga O (1957) Origin of the European cultivated carrot. Instituut voor de Veredeling van Tuinbouwgewassen

[CR4] Bélanger S, Esteves P, Clermont I (2016). Genotyping-by-sequencing on pooled samples and its use in measuring segregation bias during the course of androgenesis in barley. Plant Genome.

[CR5] Berro I, Lado B, Nalin RS (2019). Training population optimization for genomic selection. Plant Genome.

[CR6] Brachi B, Morris GP, Borevitz JO (2011). Genome-wide association studies in plants: the missing heritability is in the field. Genome Biol.

[CR7] Bradbury PJ, Zhang Z, Kroon DE (2007). TASSEL: Software for association mapping of complex traits in diverse samples. Bioinformatics.

[CR8] Brainard SH, Bustamante JA, Dawson JC (2021). A digital image-based phenotyping platform for analyzing root shape attributes in carrot. Front Plant Sci..

[CR9] Browning BL, Zhou Y, Browning SR (2018). A one-penny imputed genome from next-generation reference panels. Am J Hum Genet.

[CR10] Caballero A, Tenesa A, Keightley PD (2015). The nature of genetic variation for complex traits revealed by GWAS and regional heritability mapping analyses. Genetics.

[CR11] Castelain M, Le Hir R, Bellini C (2012). The non-DNA-binding bHLH transcription factor PRE3/bHLH135/ATBS1/TMO7 is involved in the regulation of light signaling pathway in Arabidopsis. Physiol Plant.

[CR12] Corak KE, Ellison SL, Simon PW (2019). Comparison of representative and custom methods of generating core subsets of a carrot germplasm collection. Crop Sci.

[CR13] Corak KE (2021). Strategies to identify and introgress production and quality traits from genetic resources to elite carrot cultivars.

[CR14] Daetwyler HD, Kemper KE, van der Werf JHJ, Hayes BJ (2012). Components of the accuracy of genomic prediction in a multi-breed sheep population1. J Anim Sci.

[CR15] Driscoll MK, McCann C, Kopace R (2012). Cell shape dynamics: from waves to migration. PLoS Comput Biol.

[CR16] Edwards SM, Buntjer JB, Jackson R (2019). The effects of training population design on genomic prediction accuracy in wheat. Theor Appl Genet.

[CR17] Elhiti M, Stasolla C (2009). Structure and function of homodomain-leucine zipper (HD-Zip) proteins. Plant Signal Behav.

[CR18] Ellison SL, Luby CH, Corak KE (2018). Carotenoid presence is associated with the Or gene in domesticated carrot. Genetics.

[CR19] Endelman JB (2011). Ridge regression and other kernels for genomic selection with R package rrBLUP. Plant Genome.

[CR20] Endelman JB, Jannink J-L (2012). Shrinkage estimation of the realized relationship matrix. G3 Bethesda.

[CR21] Erbe M, Gredler B, Seefried FR (2013). A function accounting for training set size and marker density to model the average accuracy of genomic prediction. PLoS ONE.

[CR22] Ewens WJ, Spielman RS (1995). The transmission/disequilibrium test: history, subdivision, and admixture. Am J Hum Genet.

[CR23] FAO (2020) Food and Agriculture Organization of the United Nations statistics database. Rome, Italy: FAO. http://www.fao.org/faostat/en/#data

[CR24] Hadasch S, Simko I, Hayes RJ (2016). Comparing the predictive abilities of phenotypic and marker-assisted selection methods in a biparental lettuce population. Plant Genome.

[CR25] Henderson CR (1973). Sire evaluation and genetic trends. J Anim Sci.

[CR26] Henderson CR (1963) Selection index and expected genetic advance. Statisitical Genet Plant Breed

[CR27] Hernandez CO, Wyatt LE, Mazourek MR (2020) Genomic prediction and selection for fruit traits in winter squash. G3 (Bethesda) 10:3601–3610. 10.1534/g3.120.401215 10.1534/g3.120.401215 PMC7534422 32816923

[CR28] Iorizzo M, Ellison S, Senalik D (2016). A high-quality carrot genome assembly provides new insights into carotenoid accumulation and asterid genome evolution. Nat Genet.

[CR29] Iorizzo M, Bostan H, Ellison S, et al (2020) Improved hybrid de novo genome assembly, gene prediction and annotation of carrot (Daucus carota). Plant and Animal Genome Proceedings: Apiaceae Workshop. San Diego, CA, January, 2020

[CR30] Kang HM, Zaitlen NA, Wade CM (2008). Efficient control of population structure in model organism association mapping. Genetics.

[CR31] Lipka AE, Tian F, Wang Q (2012). GAPIT: genome association and prediction integrated tool. Bioinformatics.

[CR32] Liu X, Huang M, Fan B (2016). Iterative usage of fixed and random effect models for powerful and efficient genome-wide association studies. PLoS Genet.

[CR33] Lozada DN, Mason RE, Sarinelli JM, Brown-Guedira G (2019). Accuracy of genomic selection for grain yield and agronomic traits in soft red winter wheat. BMC Genet.

[CR34] Macko-Podgórni A, Stelmach K, Kwolek K (2020). Mining for candidate genes controlling secondary growth of the carrot storage root. Int J Mol Sci.

[CR35] Meuwissen THE, Hayes BJ, Goddard ME (2001). Prediction of total genetic value using genome-wide dense marker maps. Genetics.

[CR36] Minamikawa MF, Takada N, Terakami S (2018). Genome-wide association study and genomic prediction using parental and breeding populations of Japanese pear (Pyrus pyrifolia Nakai). Sci Rep.

[CR37] Mousavi SAR, Dubin AE, Zeng W-Z (2021). PIEZO ion channel is required for root mechanotransduction in Arabidopsis thaliana. Proc Natl Acad Sci.

[CR38] Myles S, Peiffer J, Brown PJ (2009). Association mapping: critical considerations shift from genotyping to experimental design. Plant Cell.

[CR39] Olatoye MO, Clark LV, Labonte NR, et al (2020) Training population optimization for genomic selection in miscanthus. G3 (Bethesda) 10:2465–2476. 10.1534/g3.120.401402 10.1534/g3.120.401402 PMC7341128 32457095

[CR40] Otyama PI, Wilkey A, Kulkarni R, et al (2019) Evaluation of linkage disequilibrium, population structure, and genetic diversity in the U.S. peanut mini core collection. BMC Genomics 20:481. 10.1186/s12864-019-5824-9 10.1186/s12864-019-5824-9 PMC6558826 31185892

[CR41] Price AL, Patterson NJ, Plenge RM (2006). Principal components analysis corrects for stratification in genome-wide association studies. Nat Genet.

[CR42] Pritchard JK, Rosenberg NA (1999). Use of unlinked genetic markers to detect population stratification in association studies. Am J Hum Genet.

[CR43] Pritchard JK, Stephens M, Donnelly P (2000). Inference of population structure using multilocus genotype data. Genetics.

[CR44] R Core Team (2021) R: A Language and Environment for Statistical Computing. R Foundation for Statistical Computing, Vienna, Austria. https://www.R-project.org

[CR45] Riedelsheimer C, Endelman JB, Stange M (2013). Genomic predictability of interconnected biparental maize populations. Genetics.

[CR46] Robinson MR, Wray NR, Visscher PM (2014). Explaining additional genetic variation in complex traits. Trends Genet.

[CR47] Rogers AR, Huff C (2009). Linkage disequilibrium between loci with unknown phase. Genetics.

[CR48] Rosyara UR, De Jong WS, Douches DS, Endelman JB (2016). Software for genome-wide association studies in autopolyploids and its application to potato. Plant Genome.

[CR50] Simon PW (2000) Domestication, historical development, and modern breeding of carrot. In: Plant Breeding Reviews. John Wiley & Sons, Ltd, pp 157–190

[CR51] Simon PW, Freeman RE, Vieira JV, Prohens J, Nuez F (2008). Carrot - vegetables ii: Fabaceae, Liliaceae, Solanaceae, and Umbelliferae. Springer.

[CR52] Srivastava RK, Singh RB, Pujarula VL (2020). Genome-wide association studies and genomic selection in pearl millet: advances and prospects. Front Genet.

[CR53] Sverrisdóttir E, Sundmark EHR, Johnsen HØ (2018). The value of expanding the training population to improve genomic selection models in tetraploid potato. Front Plant Sci.

[CR54] Tayeh N, Klein A, Le Paslier M-C (2015). Genomic prediction in pea: effect of marker density and training population size and composition on prediction accuracy. Front Plant Sci.

[CR55] Tsai H-Y, Janss LL, Andersen JR (2020). Genomic prediction and GWAS of yield, quality and disease-related traits in spring barley and winter wheat. Sci Rep.

[CR56] Turner SD, Senalik DA, Simon PW (2018). An automated image analysis pipeline enables genetic studies of shoot and root morphology in carrot (Daucus carota L.). Front Plant Sci.

[CR57] VanRaden PM (2008). Efficient methods to compute genomic predictions. J Dairy Sci.

[CR58] Wang K, He J, Zhao Y (2018). EAR1 negatively regulates ABA signaling by enhancing 2C protein phosphatase activity. Plant Cell.

[CR59] Wang Q, Yu Y, Yuan J (2017). Effects of marker density and population structure on the genomic prediction accuracy for growth trait in Pacific white shrimp Litopenaeus vannamei. BMC Genet.

[CR60] Wu X-L, Xu J, Feng G (2016). Optimal design of low-density SNP arrays for genomic prediction: algorithm and applications. PLoS ONE.

[CR61] Xu X, Ji J, Xu Q (2018). The major-effect quantitative trait locus CsARN6.1 encodes an AAA ATPase domain-containing protein that is associated with waterlogging stress tolerance by promoting adventitious root formation. Plant J.

[CR62] Xu Y, Wang X, Ding X (2018). Genomic selection of agronomic traits in hybrid rice using an NCII population. Rice.

[CR63] Yu J, Pressoir G, Briggs WH (2006). A unified mixed-model method for association mapping that accounts for multiple levels of relatedness. Nat Genet.

[CR64] Zhang A, Wang H, Beyene Y (2017). Effect of trait heritability, training population size and marker density on genomic prediction accuracy estimation in 22 bi-parental tropical maize populations. Front Plant Sci.

[CR65] Zhang X, Pérez-Rodríguez P, Semagn K (2015). Genomic prediction in biparental tropical maize populations in water-stressed and well-watered environments using low-density and GBS SNPs. Heredity (edinb).

[CR66] Zhang Z, Ersoz E, Lai CQ (2010). Mixed linear model approach adapted for genome-wide association studies. Nat Genet.

