# Supplementary material for: Genetic characterization of carrot root shape and size using genome-wide association analysis and genomic-estimated breeding values
Source: Theor Appl Genet. 2021 Nov 15;135(2):605–22. doi: 10.1007/s00122-021-03988-8 (PMC8866378; doi:10.1007/s00122-021-03988-8)
Supplement: Supplementary file 1 — Supplementary file1 (DOCX 26 kb) [file 122_2021_3988_MOESM1_ESM.docx]

**Supplementary Table 1.** Candidate genes in close physical proximity to SNPs identified in the GWAS analyses. 40 kb windows around each significant SNP were selected based on the attainment of linkage equilibrium (at *r*^2^ = 0.1) at a distance of 37.1 kb between SNP markers.

| **Trait** | **SNP position (chromosome:bp)** | **Gene ID** | **Gene position (bp)** | **Description** |
| --- | --- | --- | --- | --- |
| *Root fill* | DCv3_Chr2:47341762 | DCv3_Chr2.08062 | 47341293 - 47342726 | AAA-ATPase At3g28510-like |
|  |  | DCv3_Chr2.08061 | 47332912 - 47333562 | Transcription factor bHLH135 family |
|  |  | DCv3_Chr2.08063 | 47343834 - 47344832 | AAA-ATPase At3g28510-like |
|  |  | DCv3_Chr2.08064 | 47347372 - 47348712 | F-box/kelch-repeat protein SKIP11-like |
|  |  | DCv3_Chr2.08059 | 47320676 - 47322316 | homeobox-leucine zipper protein ATHB-13-like |
|  |  | DCv3_Chr2.08060 | 47326435 - 47328431 | binding partner of ACD11 1 |
|  |  | DCv3_Chr2.08065 | 47355375 - 47355929 | protein transport-IEA, predicted/uncharacterized |
|  |  | DCv3_Chr2.08066 | 47357387 - 47358874 | pentatricopeptide repeat-containing protein At1g26900 |
|  |  | DCv3_Chr2.08067 | 47359227 - 47366633 | Ultraviolet-B receptor like |
| *Max width* | DCv3_Chr3:58042921 | DCv3_Chr3.13679 | 58025675 - 58029971 | proline-rich receptor-like protein kinase PERK1 isoform X2 |
|  |  | DCv3_Chr3.13680 | 58036126 - 58039097 | chlorophyll(ide) b reductase NYC1 protein |
|  |  | DCv3_Chr3.13681 | 58041494 - 58043511 | F-box/LRR-repeat/kelch-repeat protein At2g27520-like |
|  |  | DCv3_Chr3.13682 | 58045628 - 5804593 | F-box/LRR-repeat/kelch-repeat protein At2g27520-like |
|  |  | DCv3_Chr3.13683 | 58047937 - 58048719 | transcription repressor OFP8-like |
|  |  | DCv3_Chr3.13684 | 58054490 - 58055101 | predicted/uncharacterized |
|  | DCv3_Chr7:6622754 | DCv3_Chr7.27425 | 6602567 - 6607204 | extensin-2-like |
|  |  | DCv3_Chr7.27426 | 6612588 - 6624303 | clathrin interactor EPSIN 3 isoform X1 |
|  |  | DCv3_Chr7.27427 | 6630376 - 6648842 | precorrin-4 C11-methyltransferase activity-IEA |
| *Length* | DCv3_Chr2:42684849 | DCv3_Chr2.07411 | 42663991 - 42668288 | kDa heat- and acid-stable phosphoprotein isoform X2 |
|  |  | DCv3_Chr2.07412 | 42670014 - 42672653 | WUSCHEL-related homeobox 9-like isoform X2 |
|  |  | DCv3_Chr2.07413 | 42681217 - 42681762 | pathogenesis-related genes transcriptional activator PTI5-like |
|  |  | DCv3_Chr2.07414 | 42684371 - 42685126 | ethylene-responsive transcription factor ERF091 [Rosa chinensis] PRQ29060.1 putative transcription factor AP2-EREBP family |
|  |  | DCv3_Chr2.07415 | 42687057 - 42688797 | GDSL esterase/lipase At5g45950 |
|  |  | DCv3_Chr2.07416 | 42690537 - 42693526 | myosin-light-chain-phosphatase activity-ISS |
|  |  | DCv3_Chr2.07417 | 42699797 - 42710180 | apoptosis inhibitor 5-like protein API5 |
|  | DCv3_Chr5:34380903 | DCv3_Chr5.21023 | 34359807 - 34377747 | piezo-type mechanosensitive ion channel homolog isoform X2 |
|  |  | DCv3_Chr5.21024 | 34379606 - 34381171 | protein RESISTANCE TO PHYTOPHTHORA 1 |
|  |  | DCv3_Chr5.21025 | 34388288 - 34389865 | transcription termination factor MTERF4 |
|  |  | DCv3_Chr5.21026 | 34393352 - 34396024 | multiple organellar RNA editing factor 8 |
|  |  | DCv3_Chr5.21027 | 34399058 - 34401976 | predicted/uncharacterized |
| *L/W ratio* | DCv3_Chr3:7295883 | DCv3_Chr3.10642 | 7276426 - 7278018 | gibberellin 20 oxidase 1-D-like |
|  |  | DCv3_Chr3.10643 | 7285015 - 7286955 | receptor-like serine/threonine-protein kinase At4g25390 |
|  |  | DCv3_Chr3.10644 | 7295109 - 7297436 | subtilisin-like protease SBT1.3 |
|  |  | DCv3_Chr3.10645 | 7305488 - 7306843 | 3-ketoacyl-CoA synthase 5-like |
|  | DCv3_Chr3:56902806 | DCv3_Chr3.13539 | 56881286 - 56885945 | beta-galactosidase-like isoform X2 |
|  |  | DCv3_Chr3.13540 | 56893175 - 56897187 | beta-galactosidase-like |
|  |  | DCv3_Chr3.13541 | 56901066 - 56905107 | beta-galactosidase-like |
|  |  | DCv3_Chr3.13542 | 56908315 - 56913024 | beta-galactosidase-like |
|  | DCv3_Chr4:26702749 | DCv3_Chr4.15205 | 26688512 - 26688946 | serine/threonine-protein phosphatase 7 long form homolog isoform X4 |
|  |  | DCv3_Chr4.15206 | 26715373 - 26715648 | pentatricopeptide repeat-containing protein At3g02330 |
|  |  | DCv3_Chr4.15207 | 26719838 - 26721154 | putative plasma membrane intrinsic protein |
|  | DCv3_Chr4:33224217 | DCv3_Chr4.15955 | 33212022 - 33214334 | pentatricopeptide repeat-containing protein At4g32430 |
|  |  | DCv3_Chr4.15956 | 33215011 - 33217896 | signal peptidase complex catalytic subunit SEC11A-like |
|  |  | DCv3_Chr4.15957 | 33221585 - 33222652 | chaperone protein dnaJ 49-like |
|  |  | DCv3_Chr4.15958 | 33224330..33228140 | ribosome biogenesis regulatory protein homolog |
|  |  | DCv3_Chr4.15959 | 33230293 - 33233567 | Adenine nucleotide transporter like |
|  |  | DCv3_Chr4.15960 | 33235865 - 33237879 | uncharacterized/predicted (At2g34160-like) |
|  |  | DCv3_Chr4.15961 | 33239808 - 33243872 | DEAD-box ATP-dependent RNA helicase 52C-like |
|  | DCv3_Chr5:39853263 | DCv3_Chr5.21650 | 39833046 - 39834587 | ATP-dependent RNA helicase DEAH13-like isoform X1 |
|  |  | DCv3_Chr5.21651 | 39834690 - 39835832 | Translation initiation factor 3 |
|  |  | DCv3_Chr5.21652 | 39836944 - 39837219 | RALF-like 7 |
|  |  | DCv3_Chr5.21653 | 39845919 - 39847859 | pentatricopeptide repeat-containing protein At3g46790 |
|  |  | DCv3_Chr5.21654 | 39849050 - 39851083 | serine/threonine-protein phosphatase PP1 isozyme 2-like |
|  |  | DCv3_Chr5.21655 | 39853532 - 39855636 | ras-related protein Rab2BV-like |
|  |  | DCv3_Chr5.21656 | 39859858 - 39861877 | predicted/uncharacterized |
|  |  | DCv3_Chr5.21657 | 39865612 - 39867210 | UDP-glucose iridoid glucosyltransferase-like isoform X2 |
|  |  | DCv3_Chr5.21658 | 39868096 - 39869725 | UDP-glucose iridoid glucosyltransferase-like |
|  |  | DCv3_Chr5.21659 | 39871553 - 39874141 | Bifunctional lysine-specific demethylase and histidyl-hydroxylase |
|  | DCv3_Chr6:37411769 | DCv3_Chr6.26225 | 37390092 - 37392430 | arginine/serine-rich coiled-coil protein 2-like |
|  |  | DCv3_Chr6.26226 | 37396326 - 37401019 | DEAD-box ATP-dependent RNA helicase 7-like |
|  |  | DCv3_Chr6.26227 | 37410135 - 37412447 | subtilisin-like protease SBT1.3 |
|  |  | DCv3_Chr6.26228 | 37412939 - 37414331 | PTI1-like tyrosine-protein kinase At3g15890 |
|  |  | DCv3_Chr6.26229 | 37415459 - 37417180 | PTI1-like tyrosine-protein kinase At3g15890 |
|  |  | DCv3_Chr6.26230 | 37418419 - 37422291 | probable fructokinase-7 |
|  |  | DCv3_Chr6.26231 | 37423639 - 37427179 | predicted/uncharacterized |
|  |  | DCv3_Chr6.26232 | 37428378 - 37432473 | peptidyl-prolyl cis-trans isomerase FKBP53-like isoform X2 |
|  | DCv3_Chr9:45393500 | DCv3_Chr9.36165 | 45374796 - 45375161 | protein SIEVE ELEMENT OCCLUSION B-like isoform X2 |
|  |  | DCv3_Chr9.36166 | 45385872 - 45388486 | protein terminal ear1 homolog |
|  |  | DCv3_Chr9.36167 | 45391751 - 45394979 | D-cysteine desulfhydrase 2 |
|  |  | DCv3_Chr9.36168 | 45397357 - 45402284 | actin-related protein 3 |
|  |  | DCv3_Chr9.36169 | 45411562 - 45413499 | cleavage and polyadenylation specificity factor subunit CG7185 |
