# Supplementary material for: Genetic characterization of carrot root shape and size using genome-wide association analysis and genomic-estimated breeding values
Source: Theor Appl Genet. 2021 Nov 15;135(2):605–22. doi: 10.1007/s00122-021-03988-8 (PMC8866378; doi:10.1007/s00122-021-03988-8)
Supplement: Supplementary file 2 — Supplementary file2 (DOCX 19 kb) [file 122_2021_3988_MOESM2_ESM.docx]

**Supplementary Table 2.** GEBVs for the seven carrot root traits listen in Table 2. The entire diversity panel of 662 carrot accessions was utilized for phenotyping, but only SNPs located on chromosome 3 were used in estimating genetic variance.

| **Trait** | ***h*^2^** | **Pred. accur. (avg)** | **Pred. accur. (min)** |
| --- | --- | --- | --- |
| *Shoulder curvature* | 0.62 | 0.53 | 0.14 |
| *Tip curvature* | 0.04 | 0.09 | 0.07 |
| *Root fill* | 0.62 | 0.77 | 0.07 |
| *Biomass* | 0.36 | 0.38 | 0.10 |
| *Max width* | 0.61 | 0.65 | 0.08 |
| *Length* | 0.65 | 0.68 | 0.17 |
| *L/W ratio* | 0.80 | 0.72 | 0.15 |
