# Supplementary figures and images for: Genetic characterization of carrot root shape and size using genome-wide association analysis and genomic-estimated breeding values
Source: Theor Appl Genet. 2021 Nov 15;135(2):605–22. doi: 10.1007/s00122-021-03988-8 (PMC8866378; doi:10.1007/s00122-021-03988-8)

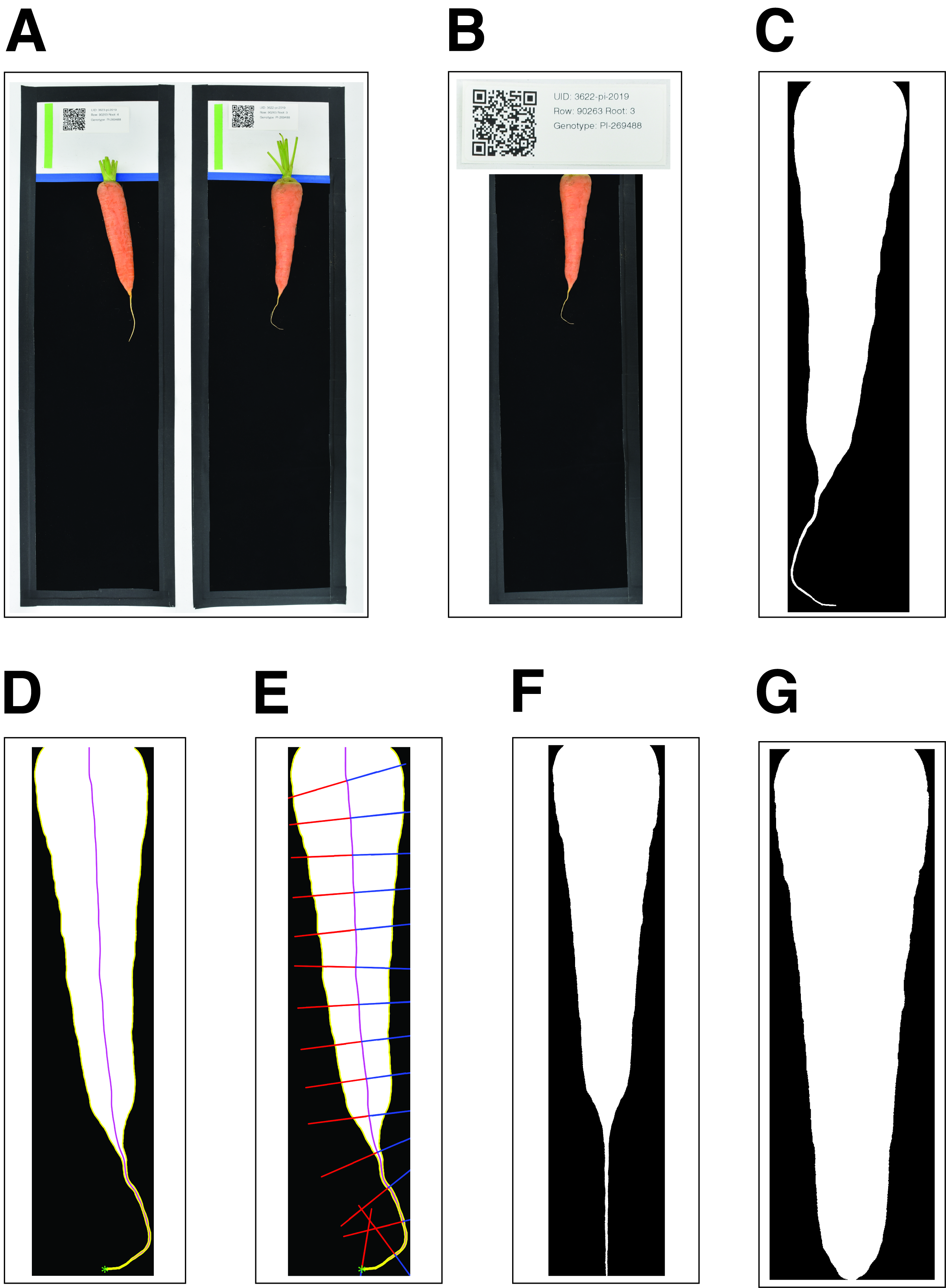

Supplement: Supplementary file 3 — Supplementary Fig. 1 Image acquisition workflow (adapted from Brainard et al. 2021). A Each black-bordered box within the overall image is first identified; B QR codes in the upper portion of each sub-box were scanned; C Carrot pixels were differentiated from background pixels to generate binary mask images; D Binary masks were saved to disk according to a file structure and naming scheme based on the information encoded within each QR code. E The midline of the carrot root was estimated by tracing a path from the carrot tip to the center of the shoulder, following the maximum of the smoothed Euclidean distance transform; Fdth measurements were made by sampling the binary mask normal to vectors tangent to points along the midline; G-HA random forest classifier was used to detect the point at which to “de-tip” any residual, unexpanded portion of the tap root [file 122_2021_3988_MOESM3_ESM.tif]
